# A Psychological Network Approach to Attitudes and Preventive Behaviors During Pandemics: A COVID-19 Study in the United Kingdom and the Netherlands

**DOI:** 10.1177/19485506211002420

**Published:** 2022-01

**Authors:** Monique Chambon, Jonas Dalege, Janneke E. Elberse, Frenk van Harreveld

**Affiliations:** 1 10206 National Institute for Public Health and the Environment (RIVM), Bilthoven, the Netherlands; 2 Department of Social Psychology, 1234 University of Amsterdam, the Netherlands; 3 Santa Fe Institute, NM, USA

**Keywords:** COVID-19, pandemic, preventive behaviors, compliance, complex systems

## Abstract

Preventive behaviors are crucial to prevent the spread of the coronavirus causing COVID-19. We adopted a complex psychological systems approach to obtain a descriptive account of the network of attitudes and behaviors related to COVID-19. A survey study (*N =* 1,022) was conducted with subsamples from the United Kingdom (*n* = 502) and the Netherlands (*n* = 520). The results highlight the importance of people’s support for, and perceived efficacy of, the measures and preventive behaviors. This also applies to the perceived norm of family and friends adopting these behaviors. The networks in both countries were largely similar but also showed notable differences. The interplay of psychological factors in the networks is also highlighted, resulting in our appeal to policy makers to take complexity and mutual dependence of psychological factors into account. Future research should study the effects of interventions aimed at these factors, including effects on the network, to make causal inferences.

During the COVID-19 pandemic, declared as such in March 2020, many public health agencies published behavioral guidelines for the public to prevent the spread of the virus (Centers for Disease Control and Prevention [CDC], 2020; National Institute for Public Health and the Environment [RIVM], 2020; World Health Organization [WHO], 2020). The recommended preventive behaviors were generally consistent; hygiene behaviors (e.g., wash hands frequently), social distancing (i.e., keep a safe distance from others), and stricter recommendations for those with symptoms. Translation of guidelines into governmental policies varied considerably between countries during the beginning of the pandemic. For example, in the Netherlands (NL), there was an “intelligent lockdown” (i.e., the population was strongly *advised* to stay home), whereas countries such as the United Kingdom (UK) and France implemented a stricter policy, that is, a total lockdown. In either case, the effectiveness of the behavioral guidelines in limiting the spread of the virus depends on the degree to which people (are willing to) adopt them (Betsch et al., 2020).

Research into pandemics shows that the degree to which people adopt preventive behaviors varies (Van et al., 2010; Webster et al., 2020). The present research provides a descriptive account of the network of attitudes and behaviors related to COVID-19. In doing so, we shed light onto which psychological factors related to adoption of the preventive behaviors are most closely related and whether differences between two countries can be observed. To do so, we use network models that illuminate the complex interplay of factors. This research provides a useful starting point to inform policy, by providing directions for future research into interventions to stimulate adoption of preventive behaviors during a pandemic.

## Adopting Preventive Behaviors in Pandemics

In determining which psychological factors likely relate to the adoption of preventive behaviors during the COVID-19 pandemic, we first examine more general factors related to health behaviors (e.g., Conner & Sparks, 2005). Two prevalent frameworks within this literature are the theory of planned behavior (TPB; Ajzen, 1991) and the health belief model (HBM; Rosenstock, 1966). The TPB explains behavior through behavioral intention, which is determined by attitudes, perceived control, and social norms. The HBM explains health-related behavior through perceived threat of the health risk (risk susceptibility and severity), costs and benefits of the preventive behavior, self-efficacy, and cues to action.

Attitudes are known to be key predictors of behavior during pandemics (Bish & Michie, 2010). Examples of cognitive and affective elements of attitudes related to adopting preventive behaviors during pandemics are risk perception and beliefs regarding the efficacy of the preventive behaviors (Asmundson & Taylor, 2020; Bults et al., 2011; Plohl & Musil, 2021) and emotions such as fear and worry (Brouard et al., 2020; Harper et al., 2020), respectively. Furthermore, perceived behavioral control (Bish & Michie, 2010; Bults et al., 2011) and social norms (van Rooij et al., 2020; Webster et al., 2020) are known to influence adoption of preventive behaviors during pandemics.

While the TPB and HBM frameworks cover various key factors, their striving for a parsimonious and causal explanation of behavior requires omitting psychological variables that are relevant in practice. Other factors such as trust in authorities (Bish & Michie, 2010; Chuang et al., 2015), personality characteristics (Carvalho et al., 2020; Zettler et al., 2020), and demographics (de Zwart et al., 2010; Park et al., 2010) are also important for understanding preventive behaviors during pandemics. Furthermore, extensive literature on the effects of pandemics on physical and mental health (Leigh-Hunt et al., 2017; Wang et al., 2020), for instance due to social isolation, illustrates the importance of including health factors when studying behavior. A characteristic of the TPB and HBM frameworks is that they predominantly focus on the unidirectional effects of factors on behaviors. Given the novelty of the COVID-19 pandemic, an approach that is agnostic about the direction of relations is, in our view, more appropriate. We therefore employ a complexity approach to shed light onto factors related to preventive behaviors during the COVID-19 pandemic.

## Complex Systems Approach

Capturing the complexity of preventive behaviors in the COVID-19 pandemic requires covering a wide range of factors and their mutual dependence—a complex systems approach. The essence of such an approach is that understanding the whole system, and the mutual dependence of its elements, provides more insight than only understanding its individual elements (Luke & Stamatakis, 2012, p. 357). The value of complex systems approaches is recognized in different fields of research (Ladyman et al., 2013), including public health and global pandemics (Luke & Stamatakis, 2012). An approach suitable in the present context is provided by the Causal Attitude Network (CAN) model (Dalege et al., 2016), which conceptualizes attitudes, a key factor of interest in the current study, as complex systems.

**Figure 1.**
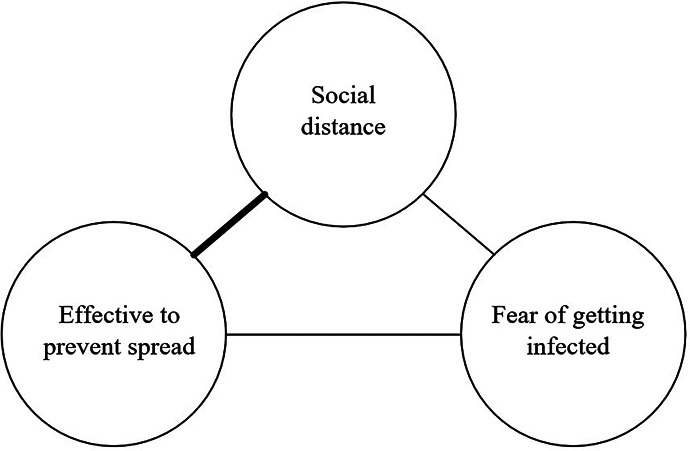
Simplified example of an attitude network toward social distance, with a cognitive evaluation (effective to prevent spread) and an affective evaluation (fear of getting infected). *Note*. The strength of the relations is indicated by edge width.

The CAN model conceptualizes attitudes as a network of different elements (nodes, i.e., evaluative reactions) and displays interactions between these attitude elements (edges). The nodes include cognitive, affective, and behavioral elements that form attitudes, and edges represent mutual dependencies between nodes (Dalege et al., 2016). To illustrate, Figure 1 shows a simplified example of a small network. Such networks provide a deeper understanding of attitudes without prior expectations about the causal relations between nodes and show the mutual dependence of the attitude elements based on empirical data.

Insight into properties of attitude networks can inform strategies to render attitude change more effective and sustainable (Dalege et al., 2019). More specifically, insight into the *connectivity* of the network provides information about the network’s strength and impact on behavior (Dalege et al., 2019). Moreover, including behavior as a node in the attitude network indicates which elements are meaningfully related to behavior (Dalege et al., 2017). Strategies aimed at *behavioral change* can be made more effective when based on such insight (Zwicker et al., 2020).

Although the CAN model provides valuable insight into the mutual dependence between attitude elements, so far it has been applied to small systems (i.e., attitudes toward a single attitude object). To optimize our understanding of adopting preventive behaviors during the unprecedented and complex COVID-19 pandemic, we expand the model’s application to other relevant factors and thus aim to further meet the complex systems objectives. We aim to demonstrate the value of such a broad approach, encompassing attitudes complemented with relevant factors based on the TPB and HBM and other relevant factors derived from the literature, such as trust, personality, and health variables.

## The Current Study

The current study has two aims. First, we aim to provide a descriptive account of the network of attitudes and behaviors related to COVID-19. In doing so, we do not limit ourselves to a set of factors in a particular model, nor do we make predictions about causality. Second, we examine whether national differences can be observed in the networks by investigating two countries with different policies: the UK (quite strict in its lockdown) and the NL (with a more advisory approach). Differences between countries in terms of their approach toward the preventive measures and promoting compliance may lead to different patterns in terms of how the psychological factors (i.e., nodes in the network) relate (see Sachisthal et al., 2019, for an example on national differences in science interest networks).

## Method

### Participants

For a moderately sized network (maximum of 30 nodes), a sample size of 500 participants is likely to result in accurate network estimation (Epskamp, 2016; van Borkulo et al., 2014), indicating that the network is representative of the population from which one samples (Epskamp et al., 2018). Due to our objective of comparing the networks of two countries, we aimed to survey a minimum of 500 participants per country. Participants were recruited via the panels of Prolific Academic in the UK (March 31 and April 1, 2020, with the exception of one participant who finished the survey April 2) and Ipsos in the NL (April 20 and April 21, 2020). Both samples were representative of their country in terms of gender and age. We included two attention checks, and participants who failed both attention checks (UK: *n* = 4, NL: *n* = 39) were excluded. This resulted in a total sample of *N* = 1,022, with subsamples of *n* = 502 from the UK and *n* = 520 from the NL (see Table 1).

**Table 1.** Demographic Information on the Total Sample and the Subsamples (UK and NL).

Variable	Total Sample	UK	NL	Difference Test
Sample size	1,022	502	520	
Gender—*n* (%) female	520 (50.9%)	257 (51.3%) ^a^	263 (50.6%)	χ^2^(1, *N* = 1,021) = .053, *p* = .818
Age range (years)	18–88	18–81	18–88	
Age mean (standard deviation)	*M* = 47.29 (*SD* = 16.03)	*M* = 45.52 (*SD* = 15.57)	*M =* 48.99 (*SD* = 16.30)	*U* = 146,168.00, *p* = .001

*Note*. UK = United Kingdom; NL = Netherlands.

^a^ One participant from the UK subsample indicated gender as *Other* and was treated as missing.

### Survey

As described in the introduction, we identified constructs in the literature that were associated with preventive behaviors during pandemics. Based on these constructs, we developed a survey (see Table 2 for item examples). Multiple items in the survey were adopted from or based on the WHO protocol for COVID-19 monitoring (WHO Regional Office for Europe, 2020).

After collecting the data, we constructed the psychological factors as nodes either with a predetermined combination of items or based on components in the data as identified through principal component analysis (PCA). The nodes on adopting behaviors (i.e., Preventive Behaviors and Repressive Behaviors) were predetermined by the behavioral guidelines as described in the next section. The node Risk Perception was the product of likelihood and severity of an infection. Other predetermined nodes were validated scales (Humanitarianism and Need for Chaos, as described below) and single-item nodes (see Table 2 for the number of items per node). The remaining nodes were constructed based on the results of the PCA (see the Online Supplemental Material, from here on referred to as Supplement, S2 for a detailed description). The survey items and scale reliability per node can also be found in the Supplement (S1 and S2). Unless otherwise specified, mean scores were calculated for the nodes consisting of more than one item. The following section provides more information on the interpretation of the nodes.

**Table 2.** Overview of the Psychological Variables (Nodes), Including the Number of Items per Node, Examples of Items, and Their Answer Scale.

Node (Items per Node)	Examples of Items per Node ( / in the Same Text Line Means Separate Item in Survey)	Scale (7-Point Likert Unless Marked ∼)
Preventive Behaviors (7)	Wash your hands frequently. / Maintain social distancing. / Stay inside the house as much as possible.	1 (*I do not display this behavior more*) to 7 (*I display this behavior much more now*)
Repressive Behaviors (3)	Stay home if you display symptoms. / Wear a face mask if you display symptoms.	
Risk Perception (2)	How likely do you believe it is you will get infected with the coronavirus within the next year?	1 (*extremely unlikely*) to 7 (*extremely likely*)
Health Risk (4)	For me personally ( / my family and friends), I consider the health risk of becoming infected with the coronavirus to be…	1 (*extremely small*) to 7 (*extremely severe*)
Consequences Society (3)	I consider the societal consequences of the corona pandemic to be…	1 (*extremely small*) to 7 (*extremely severe*)
Consequences Economy (2)	For me personally ( / my family and friends), I consider the economic consequences of the corona pandemic to be…	1 (*extremely small*) to 7 (*extremely severe*)
Affect Negative (9)	The corona pandemic makes me feel…(e.g., angry/anxious/sad/overwhelmed).	1 (*strongly disagree*) to 7 (*strongly agree*)
Affect Positive (2)	The corona pandemic makes me feel…(i.e., compassionate/grateful).	1 (*strongly disagree*) to 7 (*strongly agree*)
Worry Personal (6)	I worry about losing someone I love. / I worry about the health system being overloaded.	1 (*don’t worry at all*) to 7 (*worry a lot*)
Worry Society (3)	I worry about small companies running out of business. / I worry about a recession.	1 (*don’t worry at all*) to 7 (*worry a lot*)
Vaccination Intention (1)	If a vaccine becomes available and is recommended for me, I would get it.	1 (*strongly disagree*) to 7 (*strongly agree*)
Measures Support (3)	I support drastic measures imposed by authorities in my country to prevent the spread of the coronavirus (i.e., a lockdown).	1 (*strongly disagree*) to 7 (*strongly agree*)
Measures Efficacy (5)	I know the recommendations from authorities in my country to prevent the spread of the coronavirus. / I think the protective behaviors will limit the spread of the coronavirus.	1 (*strongly disagree*) to 7 (*strongly agree*)
Norm Society (2)	I think the majority of people display the protective behaviors.	1 (*strongly disagree*) to 7 (*strongly agree*)
Norm Family Friends (4)	I think my family and friends display the protective behaviors. / My family and friends avoid social contacts.	1 (*strongly disagree*) to 7 (*strongly agree*)
Control Infection (3)	Generally ( / For me), avoiding an infection with the coronavirus in the current situation is…	1 (*extremely difficult*) to 7 (*extremely easy*)
Trust Authorities (7)	I trust the relevant authorities in my country to adequately manage the corona pandemic. The relevant authorities in my country try hard to be fair in dealing with the corona pandemic.	1 (*strongly disagree*) to 7 (*strongly agree*)
Humanitarianism (10)	One should be kind to all people. / One should find ways to help others less fortunate than oneself.	1 (*strongly disagree*) to 7 (*strongly agree*)
Need for Chaos (8)	I get a kick when natural disasters strike in foreign countries. / There is no right and wrong in the world.	1 (*strongly disagree*) to 7 (*strongly agree*)
Health General (1)	In general, how would you rate your health?	1 (*very poor*) to 7 (*very good*) / n/a
Health Change—Physical (1)	How would you rate your physical health now as compared to before the corona pandemic?	−3 (*much worse*) to 3 (*much better*) / n/a
Health Change—Mental (1)	How would you rate your mental health now as compared to before the corona pandemic?	−3 (*much worse*) to 3 (*much better*) / n/a
Illness (1)/Smoke (1)/Age (1)/Gender (1)	Do you suffer from one or more of the following conditions? (e.g., cancer, seriously overweight)/Do you smoke?/How old are you?/What is your gender?	∼ 0 (*no*) to 1 (*yes*) / n/a */* ∼ open numeric field / ∼ 0 (*man*) to 1 (*female*) / n/a

#### Behaviors

The node on recommended behaviors was operationalized by combining the behavioral guidelines of global and national health care agencies (CDC, 2020; RIVM, 2020; WHO, 2020). This resulted in two nodes: Preventive Behaviors, consisting of items measuring the degree to which participants adopted preventive behavior (i.e., hygiene behaviors and social distancing), and Repressive Behaviors, consisting of items measuring behaviors when displaying symptoms. Higher scores indicate participants displayed the behaviors more often, or in the case of repressive behaviors would have performed them more often in case they had symptoms.

#### Attitudes

The survey contained the following scales tapping into attitudes about the pandemic relevant in the present context, divided into cognitive, affective, and behavioral factors of attitudes:

The cognitive factors of attitudes resulted in the nodes Risk Perception, Health Risk, Consequences Society, and Consequences Economy. The node Risk Perception (i.e., likelihood and severity of becoming infected) was calculated by multiplying the scores on the items.
Two nodes were identified for affect, namely Affect Negative (e.g., anger, anxiety, and confusion) and Affect Positive (i.e., compassion and gratefulness). The items on worries resulted in the node Worries Personal (e.g., getting infected, losing someone they love, or becoming lonely) for worries about events in the pandemic that affect their personal life and Worries Society (e.g., schools closing, a recession) for worries about events that affect society.
The behavioral factors of attitudes measured the intention to get vaccinated if a vaccine were available (Vaccination Intention). Attitude toward measures, including preventive behaviors, resulted in two nodes: Measures Support and Measures Efficacy, with items measuring the degree to which participants supported the measures and preventive behaviors needed to prevent the spread of the coronavirus and the degree to which they were perceived as efficient, respectively.

#### Additional factors

The social norm resulted in two nodes: Norm Society, about trusting others to (and thinking the majority does) display the preventive behaviors, and Norm Family and Friends, about the perceived degree to which family and friends adopted the preventive behaviors.

Perceived control was measured by the degree to which people felt they could avoid infection with the coronavirus, resulting in the node Control Infection.

The node Trust Authorities was measured with general trust and separate items (adopted from Mayer & Davis, 1999) for each determinant of trust (i.e., ability, benevolence, and integrity; Mayer et al., 1995).

We measured two individual variables that were expected to be a relatively stable indication of people’s response to the COVID-19 pandemic. More specifically, we expected Humanitarianism (adopted from Katz & Hass, 1988) and Need for Chaos (adopted from Petersen et al., 2018) to relate to whether people were willing to conform with the recommendations and alter their behavior, and thus accept measures for the greater good of other people’s health and a stable society, even if they themselves do not belong to a vulnerable group.

Health was measured with the single-item nodes General Health, Health Physical, and Health Mental. The latter two indicate how participants rated their physical and mental health, respectively, now compared to before the pandemic. Higher scores mean participants experienced an improvement, whereas lower scores mean they rated their health to be deteriorated. Participants also indicated whether they smoked (node Smoking) and suffered from a health condition (node Illness) known to increase the health risks of COVID-19.

The answer option *I prefer not to answer* in the health-related items and *Other* as gender, as indicated by n/a in Table 2, resulted in missing values. Twelve UK participants (2.4%) and eight NL participants (1.5%) had missing values for one or more nodes.

### Analysis

Information about preliminary and network analyses can be found in the Supplement (S2). Participants with missing values were deleted listwise due to the small number and the methodological challenges pairwise deletion would impose on the network analysis. This resulted in a final valid sample of *n* = 1,002 (UK: *n* = 490, NL: *n* = 512). The network analyses were conducted with this final valid sample.

### Social Context

**Figure 2.**
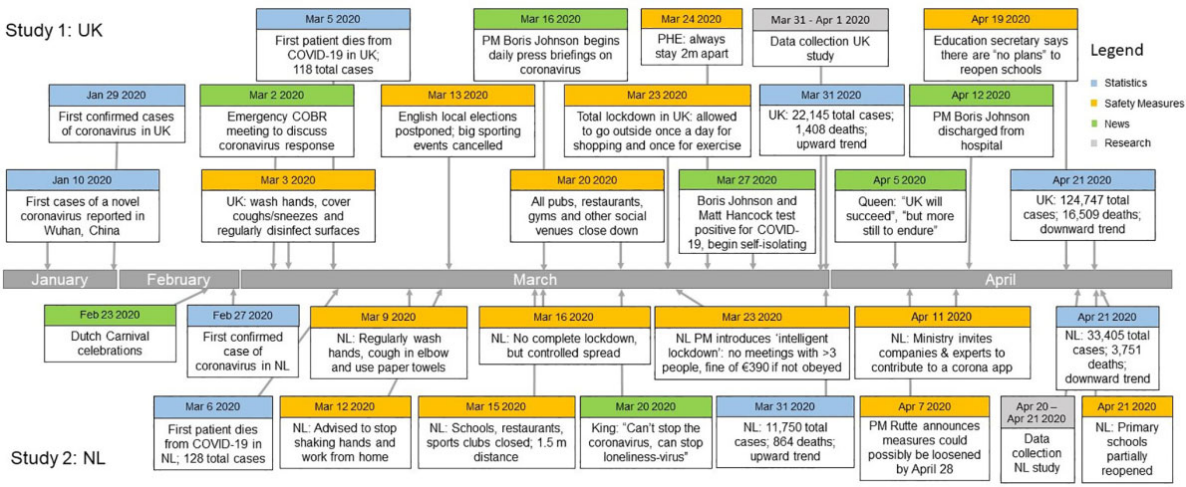
Time line of important events in the United Kingdom (upper half) and the Netherlands (lower half). *Note*. Contains relevant statistics, safety measures, and news items related to the COVID-19 pandemic, including the moments of data collection of this study.

Although it is unknown whether differences in countries’ trajectories of the pandemic affect the relations between the nodes in the networks, it is important to consider the different contexts in each of these countries at the time of data collection. Figure 2 (see S3 for underlying data) therefore shows a general time line of the UK (upper half) and the NL (lower half).

## Results

Table 3 reports the relevant statistics for each of the nodes in the network.

**Table 3.** Statistics of the Scores for All Factors (Nodes) of the Total Sample and the Subsamples With Missing Values, Including the *p* Value of Subsample Comparison Tests.

Node	Total (*N* = 1,022)			Subsample UK (*n* = 502)			Subsample NL (*n* = 520)			*p* Value
	Valid *n*	*M*	*SD*	Valid *n*	*M*	*SD*	Valid *n*	*M*	*SD*	
Preventive Behaviors	1,022	5.81	1.01	502	6.09	0.86	520	5.55	1.08	<.001*
Repressive Behaviors	1,022	4.82	1.44	502	5.18	1.26	520	4.48	1.51	<.001*
Risk Perception ^a^	1,022	17.81	9.28	502	18.29	9.44	520	17.34	9.10	.145
Health Risk	1,022	4.37	1.14	502	4.45	1.13	520	4.29	1.16	.022*
Consequences Society	1,022	5.63	1.08	502	5.73	1.00	520	5.54	1.14	.011*
Consequences Economy	1,022	4.53	1.43	502	4.89	1.43	520	4.19	1.35	<.001*
Affect Negative	1,022	3.99	1.37	502	4.35	1.38	520	3.65	1.26	<.001*
Affect Positive	1,022	4.23	1.37	502	4.35	1.39	520	4.12	1.34	.015*
Worry Personal	1,022	4.00	1.19	502	4.45	1.12	520	3.58	1.10	<.001*
Worry Society	1,022	4.64	1.28	502	4.57	1.32	520	4.70	1.24	.078
Vaccination Intention	1,022	5.94	1.58	502	6.08	1.49	520	5.80	1.64	.002*
Measures Support	1,022	5.99	1.22	502	6.48	0.89	520	5.53	1.31	<.001*
Measures Efficacy	1,022	5.93	0.79	502	6.06	0.72	520	5.81	0.83	<.001*
Norm Society	1,022	5.03	1.16	502	5.03	1.13	520	5.02	1.18	.935
Norm Family Friends	1,022	5.92	0.92	502	6.13	0.84	520	5.72	0.96	<.001*
Control Infection	1,022	4.07	1.16	502	4.02	1.18	520	4.11	1.15	.123
Trust Authorities	1,022	4.52	1.43	502	4.37	1.48	520	4.66	1.36	.002*
Humanitarianism	1,022	5.56	0.95	502	5.75	0.88	520	5.37	0.98	<.001*
Need for Chaos	1,022	1.73	0.84	502	1.77	0.82	520	1.68	0.85	.014*
Health General	1,019	5.45	1.27	500	5.42	1.29	519	5.49	1.24	.336
Health Change—Physical	1,018	4.33	1.07	500	4.32	1.04	518	4.35	1.10	.532
Health Change—Mental	1,019	3.96	1.25	501	3.74	1.26	518	4.17	1.20	<.001*
	*n*	No (%)	Yes (%)	*n*	No (%)	Yes (%)	*n*	No (%)	Yes (%)	
Illness	1,017	729 (71.7)	288 (28.3)	500	364 (72.8)	136 (27.2)	517	365 (70.6)	152 (29.4)	.436
Smoking	1,015	869 (85.6)	146 (14.4)	497	432 (86.9)	65 (13.1)	518	437 (84.4)	81 (15.6)	.246

*Note*. UK = United Kingdom; NL = Netherlands.

^a^ *This node was calculated by multiplying the scores on its two items, hence the score deviates from the other nodes.*

*Significant difference between subsamples (p *< .05).*

### Network Analyses

**Figure 3.**
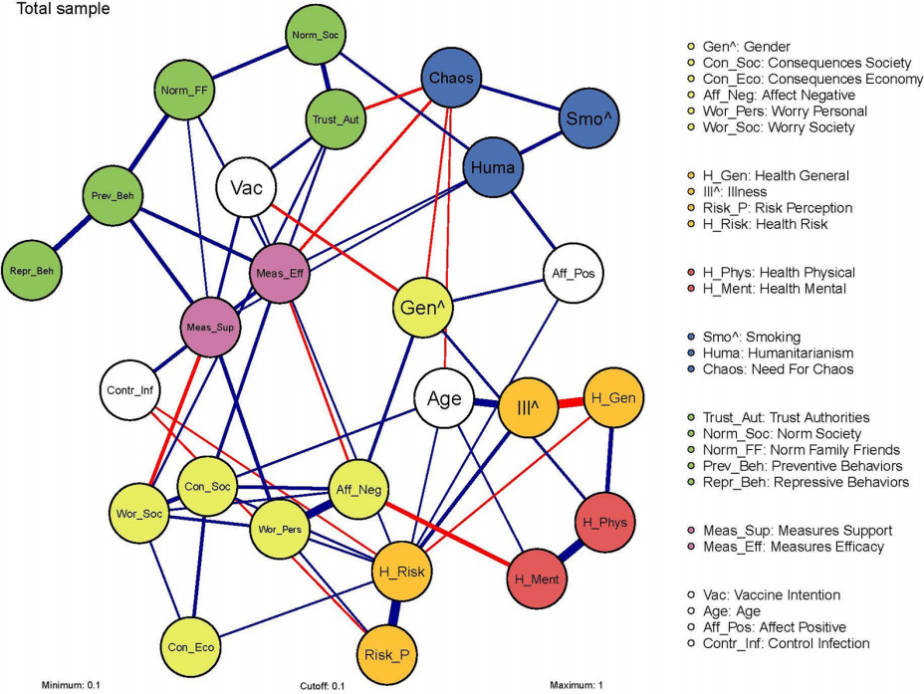
Network of attitudes and behaviors related to COVID-19. *Note*. Nodes represent the measured factors, and edges represent relations between these nodes, blue for positive and red for negative relations. Edge weights below .10 are omitted from the network to improve readability. For the binary nodes ( ^ ), a positive relationship indicates that increasing the other node results in a higher probability for Category 1 of the binary node (i.e., Gender 1 = *female*; Smoking 1 = *yes*; Illness 1 = *yes*). The strength of the relations is indicated by edge width and color density.

Figure 3 shows the network of attitudes and behaviors related to COVID-19 based on the total sample.^1^ The nodes represent the psychological factors as measured by a combination of items in the survey. The edges represent the mutual dependence between nodes. More specifically, an edge indicates a relation between two nodes after controlling for every other node in the network. These edges are weighted (i.e., the edge strength is indicated by regression weights) and undirected (i.e., the direction of the edge is undetermined). A complete overview of the edge weights for this and subsequent networks is provided in the Supplement (S2). Edge weights relevant for the edges discussed in the text are provided within parentheses.

#### Communities

The communities (i.e., clusters of nodes with the same colors) indicate a difference in interconnectedness between clusters, meaning that nodes within communities are more connected to each other than to the remaining nodes.^2^ Broadly speaking, we label the communities as social norms, trust, and behavior related to the pandemic (green); attitude toward measures and preventive behaviors (purple); worries and negative affect (yellow); general health and risk perception concerning health (orange); health change compared to before the pandemic (red); and stable, personality-based characteristics (blue). White nodes did not consistently belong to one community.

#### Nodes and relations in the network

The nodes with the strongest relation with Preventive Behaviors were Norm Family Friends (0.27), Measures Efficacy (0.20), and Measures Support (0.21). These edges were highly accurate, and there was no significant difference between the strength of these edges.^3^ This means that the relations between the nodes Preventive Behaviors with Norm Family Friends, Measures Efficacy, and Measures Support were of comparable strength, with exclusively positive relations. This indicates that, for example for the node Norm Family Friends, an increase in perceived social norm among family and friends of adopting the preventive behaviors is associated with an increase in adoption of preventive behaviors, and vice versa. The nodes Trust Authorities, Worry Personal, and Control Infection were also directly related to Preventive Behaviors (0.07, 0.06, and −0.06, respectively; omitted from Figure 3), albeit to a significantly lesser extent than the previously mentioned nodes. Interestingly, the node Norm Society was not directly related to Preventive Behaviors. Furthermore, Vaccination Intention was negatively related to Gender (−0.15) and positively related to Trust Authorities (0.16), indicating that the intention to get vaccinated is stronger among men and those reporting higher trust in those authorities responsible for managing the COVID-19 pandemic.

Regarding health, Health Mental was positively related to Health Physical (0.48) and negatively related to Affect Negative (−0.25). This indicates that an increase in mental health co-occurs with an increase in physical health and a decrease in the experience of negative emotions. Examples of relations with binary nodes are those between Illness and Age (0.41) and Illness and General Health (−0.49), indicating that increasing age or decreasing general health is associated with a higher probability of a participant reporting an illness. The caption of Figure 3 provides guidelines for further interpretation of the network.

#### Centrality measure of the nodes

Centrality measures provide information about the relative importance of nodes in the network by indicating the effect that influencing one node can have on the entire network. These measures also provide an indication of the predictive value of nodes for behavior (Dalege et al., 2017). It should, however, be noted that centrality is not an indication of causality (Dablander & Hinne, 2019). The centrality measure Strength is considered to be the most suited for psychological networks (Bringmann et al., 2019).

**Figure 4.**
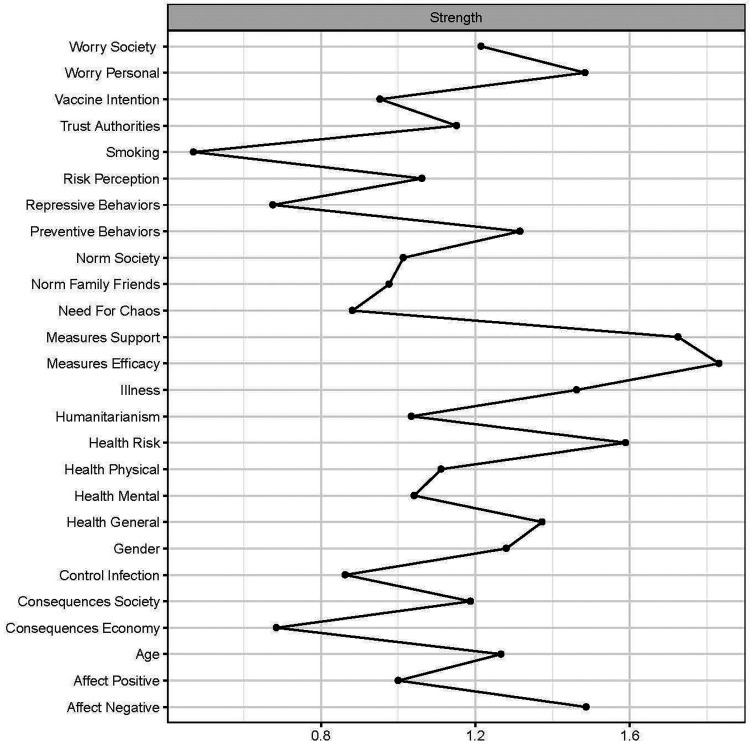
Centrality plot of the network of attitudes and behaviors related to COVID-19. *Note*. It shows the node statistic Strength, which represents the direct effect of a specific node on the network. It is calculated by the sum of the absolute edge weights of the relations a specific node has with connected nodes. A high score on Strength indicates that change in the specific node has a more profound effect on the rest of the network due to affecting many other nodes.

Figure 4 presents the centrality measure Strength^4^ (provided in text in parentheses) of the nodes in the COVID-19 network. High node strength suggests that the concerning node is an important variable for people’s attitudes and behaviors related to the COVID-19 pandemic (i.e., the COVID-19 network). The nodes relevant to Preventive Behavior (1.30), that is, Measures Efficacy, Measures Support, and Norm Family Friends, differed in strength. The node strength of Measures Efficacy (1.80) and Measures Support (1.70) was relatively high and comparable to each other (i.e., did not differ significantly). In relation to the dynamics of attitude change, high node strength suggests that changing such a node is likely to have a profound effect on the network. More specifically, it is expected to affect the interplay and mutual dependence of the psychological factors in the network, due to affecting many other nodes. However, influencing a central node will likely be difficult since it is the nature of these nodes that they are held in check by the other nodes they are connected with (Dalege et al., 2016). The node strength of Norm Family Friends (0.98) was of relatively moderate strength, although significantly lower than Measures Efficacy and Measures Support. This suggests that influencing this node is presumably feasible but will have a modest effect on the rest of the COVID-19 network. Regarding health, the nodes Health General (1.40), Health Physical (1.10), and Health Mental (1.00) showed relatively moderate strength that did not differ significantly from each other. Interestingly, Health Risk (1.60) had a significantly higher node strength than Risk Perception (1.10), indicating that change in the perceived health risk is likely to have a more profound effect on the COVID-19 network. This also applied to affect: The node Affect Negative (1.50) was significantly stronger than Affect Positive (1.00), which indicates that change in negative emotions related to the COVID-19 pandemic is likely to have more effect on the rest of the network than change in positive emotions.

### Comparing Subsamples

**Figure 5.**
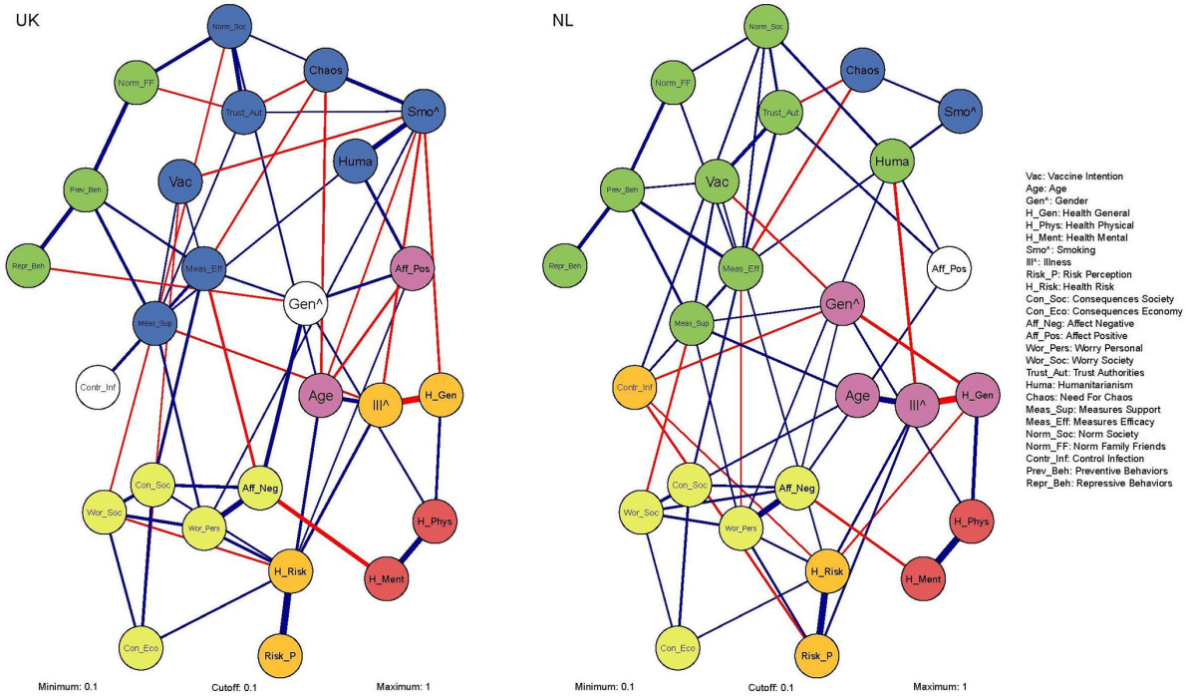
Networks of attitudes and behaviors related to COVID-19 for the subsamples of the United Kingdom (UK) and the Netherlands (NL). *Note*. See the caption of Figure 3 for guidelines on interpreting the networks.

Figure 5 shows the weighted, undirected networks of both subsamples. The communities differed between the networks of attitudes and behaviors related to COVID-19. In the UK, part of the community consisting of norms, trust, and behavior related to the pandemic found in the total sample was integrated with the community with personality-based characteristics, indicating a difference in interconnectedness between nodes. Furthermore, Measures Efficacy and Measures Support belonged to different communities depending on the subsample.

Connectivity of the networks is calculated by the average shortest path length (ASPL), indicating how quickly any given node can reach any other given node in the network on average. The ASPL of the COVID-19 networks is 11.80 for the UK subsample and 13.02 for the NL subsample. This indicates differences between the networks, but the global strength measure (a different measure of connectivity) of the Network Comparison Test (NCT) did not differ significantly (global strength = .69, *p* = .444).

**Figure 6.**
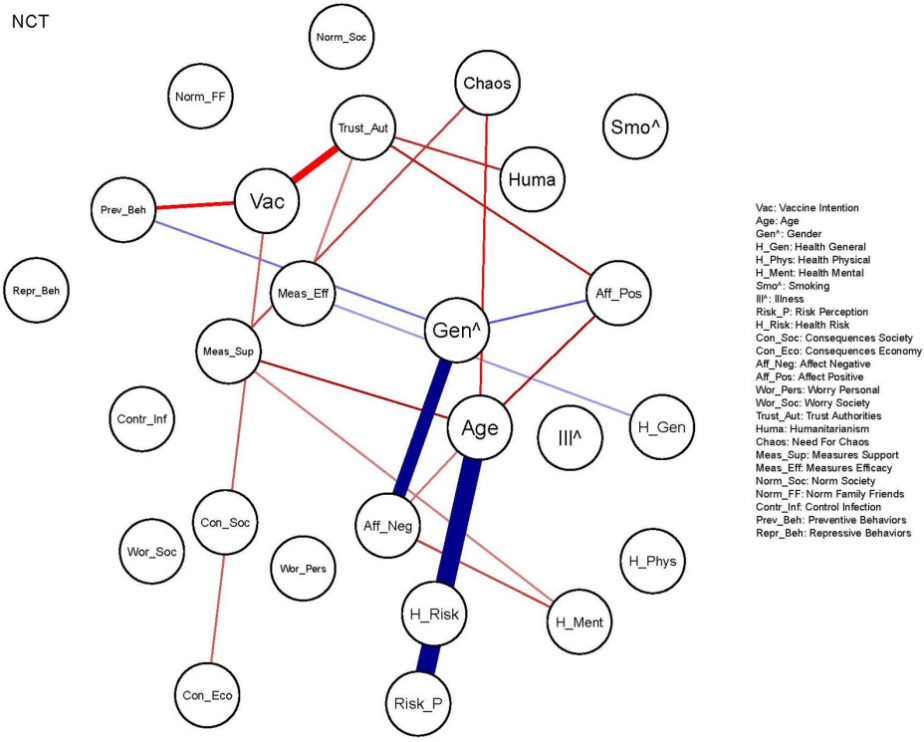
Results from the NCT, displaying edges that differ significantly between the networks of attitudes and behaviors related to COVID-19 of the UK subsample and the NL subsample. *Note*. The magnitude of the edge differences is indicated by edge width. A blue edge indicates that the relation, as indicated by the edge weight, in the NL subsample is significantly weaker, absent of (more) negative than in the UK subsample. A red edge means that the relation in the UK subsample is significantly weaker, absent of (more) negative than in the NL subsample. The edges in Figure 5 show whether the relation is positive or negative. UK = United Kingdom; NL = the Netherlands; NCT = Network Comparison Test.

Results of the NCT, aimed at formally testing differences in edges *between* networks, indicated that the edges shown in Figure 6 differed significantly between the COVID-19 networks of the subsamples. The corresponding edge weight difference, indicating the strength of the difference between the subsamples, can be found in the Supplement (S2). Edge weights relevant for the edges discussed below are provided within parentheses. The positive relation between Gender and Preventive Behaviors was only found in the UK sample (0.09). This implies that in the UK, women reported a higher degree of adopting preventive behaviors than men. Interestingly, in the NL, Vaccination Intention was positively related to Preventive Behaviors (0.11), whereas in the UK these nodes showed a negative relation (−0.05). Furthermore, the NL subsample showed a significantly stronger (positive) relation between Trust Authorities and Vaccination Intention (NL: 0.25; UK: 0.07) and Measures Efficacy (NL: 0.18; UK: 0.09), which indirectly also affects Preventive Behaviors. Regarding health, the (negative) relation between Health Mental and Affect Negative was significantly stronger in the UK (−0.29; NL: −0.16). Moreover, a (positive) relation between Health Mental and Measures Support was only found in the NL (0.08). This is relevant as it implies an indirect relation between Health Mental and Preventive Behavior, indicating that change in mental health during the pandemic and the degree to which preventive behaviors are adopted are (indirectly) related in the NL.

A salient difference in the subsamples was the relation between Age and Risk Perception, which was found solely in the UK (0.20), indicating that only there, older participants perceived more risk. The positive relation between Gender and Affect Negative also differed significantly. In both samples, women showed higher negative affect than men, but this relation was more pronounced in the UK (0.29) than in the NL (0.10).

#### Centrality measure of the nodes

**Figure 7.**
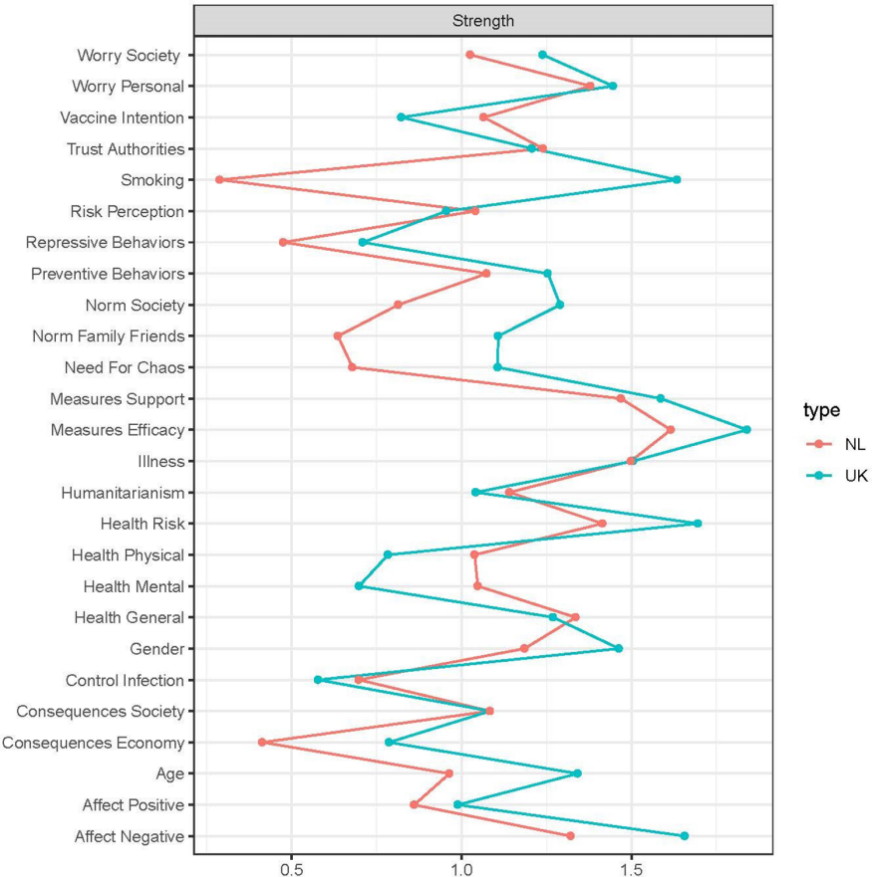
Centrality plot with node statistic Strength of the network of attitudes and behaviors related to COVID-19 for the UK subsample (green) and the NL subsample (red). *Note*. See the caption of Figure 4 for interpretation guidelines. UK = United Kingdom; NL = the Netherlands.

Figure 7 shows the centrality measure Strength of the nodes in the subsamples’ networks of attitudes and behaviors related to COVID-19. Differences between the subsamples on this measure indicate differences between countries in the relative importance of specific nodes for attitudes and behaviors related to COVID-19 in that country. The results, for instance, suggest that even though influencing the degree to which participants perceive the preventive measures as efficient (Measures Efficacy; UK: 1.80, NL: 1.60) is possibly more difficult in the UK, the effect on the rest of the COVID-19 network will likely be more profound than in the NL. The opposite seems to apply to health-related nodes: although influencing Health Physical (UK: 0.78, NL: 1.00) and Health Mental (UK: 0.70, NL: 1.00) during the pandemic is presumably more difficult in the NL, it will likely have a more profound effect on the rest of the COVID-19 network than in the UK. In general terms, the nodes related to change in health during the pandemic have a larger impact on the rest of the COVID-19 network in the NL, whereas the nodes related to social norms have a larger impact on the rest of the network in the UK.

## Discussion

The current research provides a descriptive account of the network of attitudes and behaviors related to COVID-19, thereby providing insight into the complex psychological system related to the COVID-19 pandemic. Moreover, we compared the psychological systems of two countries to explore whether differences between countries can be observed in the psychological COVID-19 networks.

First, we found that attitudes toward the measures and preventive behaviors had the strongest (positive) relation with the degree to which people adopted them. More specifically, this applied to the degree to which people support the measures and behaviors and perceive them as effective. Furthermore, we found that the perceived norm of family and friends adopting the preventive measures was also associated with adopting preventive behaviors. These findings are in line with the TPB and earlier research pointing toward attitudes and social norms as important predictors of behavior during pandemics (Bish & Michie, 2010; van Rooij et al., 2020). We complement this work by distinguishing different specifications of the known factors. This results in insights such as that the social norm of family and friends is more strongly related to adopting preventive behaviors during a pandemic than the perceived social norm in society.

Moreover, our results provide insight into how attitudes and additional factors (e.g., trust in authorities, health, and demographics) relate to each other within a network and thereby contribute to understanding attitudes and preventive behaviors related to COVID-19. The psychological networks in this study provide a first insight into how interventions aimed at one node can affect other nodes. This can be relevant for the choice of which node to target, for instance, by targeting a node that is easier with limited effect on the network, or a node that is more difficult to perturbate, but with the potential of more sustainable change in the network. In the context of the current study, the results suggest that although changing the support for, and perceived efficacy of, measures and preventive behaviors is likely to be more difficult than changing the perceived norm of family and friends, the effect on the rest of the COVID-19 network is assumed to be more profound. Future research should study interventions to affect these nodes in order to make causal inferences and examine the effect of interventions on the interplay and mutual dependence of the psychological factors in the COVID-19 network.

Second, we observed that the UK and the NL showed similar psychological networks of factors related to the COVID-19 pandemic, with some specific differences. The COVID-19 networks showed differences in clusters of nodes with higher interconnectedness, the relative importance of nodes for the rest of the network, and the relations between specific nodes. We also found that changes in health during the pandemic likely have a larger effect on the COVID-19 network in the NL than in the UK. The opposite applies to change in social norms, which will likely have a larger effect on the network in the UK. Possible explanations for these differences between the UK and the NL should be examined in future research. A potential explanation is that data collection in the UK took place during an upward trend in terms of severity of the pandemic, whereas the data in the NL were collected during a downward trend. It is, however, unknown whether differences in the COVID-19 networks are caused by a difference in timing, trajectory, policies, or media attention during the pandemic, or other cultural or preexisting differences. Despite these differences, the countries show comparable psychological networks, and future research could investigate whether the specific cross-national differences observed here are indeed meaningful.

Limitations of this study predominantly concern the boundaries of the presented networks. As is almost inherent to complex systems, we expect more nodes to be relevant to explain attitudes and preventive behaviors related to COVID-19 than included in the present study. Future research could further increase the ecological validity of the psychological networks by including other relevant nodes. Furthermore, given the duration of a pandemic, the relative contribution of nodes is assumed to fluctuate and can differ, for instance, between the initial and perseverance period or during an upward or downward trend in severity of the pandemic. Consequently, future research should shed light on the temporal dynamics of the mutual dependence of the psychological factors that influence preventive behaviors during pandemics.

## Conclusion

The objective of this research was to study the complex psychological system that are attitudes and behaviors in the COVID-19 pandemic. Accordingly, we compared the psychological networks of two countries. Based on our results, we advise to study interventions aimed at the support for, and perceived efficacy of, the measures and behaviors combating the pandemic, and the perceived norm of family and friends adopting the preventive measures. Moreover, we would like to appeal to policy makers to take mutual dependence of psychological factors into account. Finally, while the networks in both countries were largely similar, we observed some cross-national differences that can possibly inform national strategies and policies.

## Supplemental Material

Supplemental Material, sj-docx-1-spp-10.1177_19485506211002420 - A Psychological Network Approach to Attitudes and Preventive Behaviors During Pandemics: A COVID-19 Study in the United Kingdom and the Netherlands Click here for additional data file. Supplemental Material, sj-docx-1-spp-10.1177_19485506211002420 for A Psychological Network Approach to Attitudes and Preventive Behaviors During Pandemics: A COVID-19 Study in the United Kingdom and the Netherlands by Monique Chambon, Jonas Dalege, Janneke E. Elberse and Frenk van Harreveld in Social Psychological and Personality Science

## Notes

1. The network is estimated via nodewise regression. The script is available as Online Supplemental Material (from here on referred to as Supplement) (S4).

2. More information on community analysis can be found in the Supplement (S2).

3. The results of the edge accuracy analysis and the edge difference tests, testing differences in edges *within* a network, for this and subsequent networks are made available as Supplement (S5).

4. The results of the centrality stability and difference analyses (to compare between nodes) of this and subsequent networks can be found in the Supplement (S6).
