# Supplementary material for: A Psychological Network Approach to Attitudes and Preventive Behaviors During Pandemics: A COVID-19 Study in the United Kingdom and the Netherlands
Source: Soc Psychol Personal Sci. 2022 Jan;13(1):233–45. doi: 10.1177/19485506211002420 (PMC8042407; doi:10.1177/19485506211002420)
Supplement: Supplemental Material, sj-docx-1-spp-10.1177_19485506211002420 - A Psychological Network Approach to Attitudes and Preventive Behaviors During Pandemics: A COVID-19 Study in the United Kingdom and the Netherlands [file sj-docx-1-spp-10.1177_19485506211002420.docx]

A psychological network approach to attitudes and preventive behaviors during pandemics: A COVID-19 study in the United Kingdom and the Netherlands

**Supplementary Materials**

Contents

[S1 Items per node 2](#_Toc63065355)

[S2 Analyses 10](#_Toc63065356)

[2.1 Node construction 10](#_Toc63065357)

[2.2 Scale reliability per node 16](#_Toc63065358)

[2.3 Information on analyses 17](#_Toc63065359)

[2.4 Edge weights Total sample 24](#_Toc63065360)

[2.5 Edge weights UK 25](#_Toc63065361)

[2.6 Edge weights NL 26](#_Toc63065362)

[2.7 NCT weights (UK – NL) 27](#_Toc63065363)

[S3 COVID-19 Timeline UK NL 28](#_Toc63065364)

[3.1 Visual timeline 28](#_Toc63065365)

[3.2 Data The United Kingdom 29](#_Toc63065366)

[3.3 Data The Netherlands 36](#_Toc63065367)

[3.4 Trends 42](#_Toc63065368)

[S4 R script 44](#_Toc63065369)

[S5 Edge accuracy and edge difference 44](#_Toc63065370)

[S6 Centrality stability and centrality difference 44](#_Toc63065371)

[References 45](#_Toc63065372)

# S1 Items per node

The nodes were computed by calculating mean of the items, except for *Risk perception*, for which the score on the two items was multiplied.

|  | ***Nodes*** | ***Items survey*** | ***Answer scale*** |
| --- | --- | --- | --- |
| Adopting behaviors | Preventive behaviors | Wash your hands frequently. | I do not display this behaviour more 1. - I display this behaviour much more now 7. |
|  |  | Maintain social distancing. | I do not display this behaviour more 1. - I display this behaviour much more now 7. |
|  |  | Avoid touching your face. | I do not display this behaviour more 1. - I display this behaviour much more now 7. |
|  |  | Cover coughs and sneezes. | I do not display this behaviour more 1. - I display this behaviour much more now 7. |
|  |  | Stay inside the house as much as possible. | I do not display this behaviour more 1. - I display this behaviour much more now 7. |
|  |  | Clean and disinfect surfaces. | I do not display this behaviour more 1. - I display this behaviour much more now 7. |
|  |  | Stay informed and follow advice given by professionals. | I do not display this behaviour more 1. - I display this behaviour much more now 7. |
|  |  |  |  |
|  | Repressive behaviors | Stay home if you display symptoms. | I do not display this behaviour more 1. - I display this behaviour much more now 7. |
|  |  | Wear a facemask if you display symptoms. | I do not display this behaviour more 1. - I display this behaviour much more now 7. |
|  |  | If you have a fever, cough and have difficulty breathing, seek medical care early. | I do not display this behaviour more 1. - I display this behaviour much more now 7. |
|  |  |  |  |
| *Attitudes* |  |  |  |
| Cognitive factors | Risk Perception | How likely do you believe it is you will get infected with the coronavirus within the next year? | Extremely unlikely 1. - Extremely likely 7. |
|  |  | How severe do you believe contracting the coronavirus would be for you? | Not severe 1. - Very severe 7. |
|  |  |  |  |
|  | Health Risk | Generally, I consider the health risk of becoming infected with the coronavirus to be... | Extremely small 1. - Extremely severe 7. |
|  |  | For me personally, I consider the health risk of becoming infected with the coronavirus to be… | Extremely small 1. - Extremely severe 7. |
|  |  | For my family and friends, I consider the health risk of becoming infected with the coronavirus to be… | Extremely small 1. - Extremely severe 7. |
|  |  | How susceptible do you consider yourself to an infection with the coronavirus? | Not at all susceptible 1. - Very susceptible 7. |
|  |  |  |  |
|  | Consequences Society | I consider the societal consequences of the corona pandemic to be… | Extremely small 1. - Extremely severe 7. |
|  |  | I consider the corona pandemic to be a threat that may alter society for a long time. | Strongly disagree 1. - Strongly agree 7. |
|  |  | I worry about the consequences of the corona pandemic. | Strongly disagree 1. - Strongly agree 7. |
|  |  |  |  |
|  | Consequences Economy | For me personally, I consider the economic consequences of the corona pandemic to be… | Extremely small 1. - Extremely severe 7. |
|  |  | For my family and friends, I consider the economic consequences of the corona pandemic to be… | Extremely small 1. - Extremely severe 7. |
|  |  |  |  |
| Affective factors | Affect Negative | The corona pandemic makes me feel angry. | Strongly disagree 1. - Strongly agree 7. |
|  |  | The corona pandemic makes me feel anxious. | Strongly disagree 1. - Strongly agree 7. |
|  |  | The corona pandemic makes me feel sad. | Strongly disagree 1. - Strongly agree 7. |
|  |  | The corona pandemic makes me feel confused. | Strongly disagree 1. - Strongly agree 7. |
|  |  | The corona pandemic makes me feel overwhelmed. | Strongly disagree 1. - Strongly agree 7. |
|  |  | The corona pandemic makes me feel frustrated. | Strongly disagree 1. - Strongly agree 7. |
|  |  | The corona pandemic makes me feel agitated. | Strongly disagree 1. - Strongly agree 7. |
|  |  | The corona pandemic makes me feel afraid. | Strongly disagree 1. - Strongly agree 7. |
|  |  | The corona pandemic makes me feel out of control. | Strongly disagree 1. - Strongly agree 7. |
|  |  |  |  |
|  | Affect Positive | The corona pandemic makes me feel compassionate. | Strongly disagree 1. - Strongly agree 7. |
|  |  | The corona pandemic makes me feel grateful. | Strongly disagree 1. - Strongly agree 7. |
|  |  |  |  |
|  | Worry Personal | I worry about getting infected with the coronavirus. | Don't worry at all 1. - Worry a lot 7. |
|  |  | I worry about losing someone I love. | Don't worry at all 1. - Worry a lot 7. |
|  |  | I worry about the health system being overloaded. | Don't worry at all 1. - Worry a lot 7. |
|  |  | I worry about restricted access to food supplies. | Don't worry at all 1. - Worry a lot 7. |
|  |  | I worry about blackouts. | Don't worry at all 1. - Worry a lot 7. |
|  |  | I worry about becoming lonely. | Don't worry at all 1. - Worry a lot 7. |
|  |  |  |  |
|  | Worry Society | I worry about schools closing. | Don't worry at all 1. - Worry a lot 7. |
|  |  | I worry about small companies running out of business. | Don't worry at all 1. - Worry a lot 7. |
|  |  | I worry about a recession. | Don't worry at all 1. - Worry a lot 7. |
|  |  |  |  |
| Behavioral factors | Vaccination Intention | If a vaccine becomes available and is recommended for me, I would get it. | Strongly disagree 1. - Strongly agree 7. |
|  |  |  |  |
|  | Measures Support | I think it is important to practice social distancing to prevent the spread of the coronavirus. | Strongly disagree 1. - Strongly agree 7. |
|  |  | I support drastic measures imposed by authorities in my country to prevent the spread of the coronavirus (i.e. a lockdown). | Strongly disagree 1. - Strongly agree 7. |
|  |  | As a society, we should do whatever it takes to prevent the spread of the coronavirus. | Strongly disagree 1. - Strongly agree 7. |
|  |  |  |  |
|  | Measures Efficacy | I know the recommendations from authorities in my country to prevent the spread of the coronavirus. | Strongly disagree 1. - Strongly agree 7. |
|  |  | I follow the recommendations from authorities in my country to prevent the spread of the coronavirus. | Not at all 1. - Very much so 7. |
|  |  | I know how to protect myself from the coronavirus. | Not at all 1. - Very much so 7. |
|  |  | I think the protective behaviours will limit the spread of the coronavirus. | Strongly disagree 1. - Strongly agree 7. |
|  |  | I feel responsible for preventing the spread of the coronavirus by displaying the protective behaviours. | Strongly disagree 1. - Strongly agree 7. |
|  |  |  |  |
| *Additional factors* |  |  |  |
| Social norms | Norm Society | I trust others to follow the recommendations from authorities in my country to prevent the spread of the coronavirus. | Strongly disagree 1. - Strongly agree 7. |
|  |  | I think the majority of people display the protective behaviours. | Strongly disagree 1. - Strongly agree 7. |
|  |  |  |  |
|  | Norm Family Friends | I think my family and friends display the protective behaviours. | Strongly disagree 1. - Strongly agree 7. |
|  |  | I see my family and friends washing their hands frequently. | Strongly disagree 1. - Strongly agree 7. |
|  |  | My family and friends avoid social contacts. | Strongly disagree 1. - Strongly agree 7. |
|  |  | My family and friends avoid crowded areas. | Strongly disagree 1. - Strongly agree 7. |
|  |  |  |  |
| Perceived control | Control Infection | Generally, avoiding an infection with the coronavirus in the current situation is… | Extremely difficult 1 - Extremely easy 7. |
|  |  | For me, avoiding an infection with the coronavirus in the current situation is… | Extremely difficult 1 - Extremely easy 7. |
|  |  | For my family and friends, avoiding an infection with the coronavirus in the current situation is… | Extremely difficult 1 - Extremely easy 7. |
|  |  |  |  |
| Trust in authorities | Trust Authorities | I trust the relevant authorities in my country to adequately manage the corona pandemic. | Strongly disagree 1. - Strongly agree 7. |
|  |  | The relevant authorities in my country are very capable of adequately managing the corona pandemic. | Strongly disagree 1. - Strongly agree 7. |
|  |  | I feel confident about the skills of the relevant authorities in my country to adequately manage the corona pandemic. | Strongly disagree 1. - Strongly agree 7. |
|  |  | Sound principles seem to guide the policy of the relevant authorities in my country in relation to the corona pandemic. | Strongly disagree 1. - Strongly agree 7. |
|  |  | The relevant authorities in my country try hard to be fair in dealing with the corona pandemic. | Strongly disagree 1. - Strongly agree 7. |
|  |  | The relevant authorities in my country are very concerned with the welfare of people like me during the corona pandemic. | Strongly disagree 1. - Strongly agree 7. |
|  |  | The needs and desires of people like me during the corona pandemic are very important to the relevant authorities in my country. | Strongly disagree 1. - Strongly agree 7. |
|  |  |  |  |
| Individual differences | Humanitarianism | One should be kind to all people. | Strongly disagree 1. - Strongly agree 7. |
|  |  | One should find ways to help others less fortunate than oneself. | Strongly disagree 1. - Strongly agree 7. |
|  |  | A person should be concerned about the well-being of others. | Strongly disagree 1. - Strongly agree 7. |
|  |  | There should be equality for everyone--because we are all human beings. | Strongly disagree 1. - Strongly agree 7. |
|  |  | Those who are unable to provide for their basic needs should be helped by others. | Strongly disagree 1. - Strongly agree 7. |
|  |  | A good society is one in which people feel responsible for one another. | Strongly disagree 1. - Strongly agree 7. |
|  |  | Everyone should have an equal chance and an equal say in most things. | Strongly disagree 1. - Strongly agree 7. |
|  |  | Acting to protect the rights and interests of other members of the community is a major obligation for all persons. | Strongly disagree 1. - Strongly agree 7. |
|  |  | In dealing with criminals the courts should recognize that many are victims of circumstances. | Strongly disagree 1. - Strongly agree 7. |
|  |  | Prosperous nations have a moral obligation to share some of their wealth with poor nations. | Strongly disagree 1. - Strongly agree 7. |
|  |  |  |  |
|  | Need for Chaos | I get a kick when natural disasters strike in foreign countries. | Strongly disagree 1. - Strongly agree 7. |
|  |  | I fantasize about a natural disaster wiping out most of humanity such that a small group of people can start all over. | Strongly disagree 1. - Strongly agree 7. |
|  |  | I think society should be burned to the ground. | Strongly disagree 1. - Strongly agree 7. |
|  |  | When I think about our political and social institutions, I cannot help thinking ‘just let them all burn’. | Strongly disagree 1. - Strongly agree 7. |
|  |  | We cannot fix the problems in our social institutions, we need to tear them down and start over. | Strongly disagree 1. - Strongly agree 7. |
|  |  | I need chaos around me - it is too boring if nothing is going on. | Strongly disagree 1. - Strongly agree 7. |
|  |  | Sometimes I just feel like destroying beautiful things. | Strongly disagree 1. - Strongly agree 7. |
|  |  | There is no right and wrong in the world. | Strongly disagree 1. - Strongly agree 7. |
|  |  |  |  |
| Health | General health | In general, how would you rate your health? | Very poor 1. - Very good 7. / I prefer not to say |
|  | Health change Physical | How would you rate your physical health now as compared to before the corona pandemic? | Much worse -3. - Much better 3. / I prefer not to say |
|  | Health change Mental | How would you rate your mental health now as compared to before the corona pandemic? | Much worse -3. - Much better 3. / I prefer not to say |
|  | Smoke | Do you smoke? | Yes / No / I prefer not to say |
|  | Illness | Do you suffer from one or more of the following conditions?  Diabetes.  Hypertension.  Cancer.  Chronic (long-term) respiratory diseases, such as asthma, chronic obstructive pulmonary disease (COPD), emphysema or bronchitis.  Heart disease.  Chronic kidney disease.  Chronic liver disease, such as hepatitis.  Chronic neurological conditions, such as Parkinson’s disease, motor neurone disease, multiple sclerosis (MS), a learning disability or cerebral palsy diabetes.  Problems with your spleen – for example, sickle cell disease or if you have had your spleen removed.  A weakened immune system as the result of conditions such as HIV and AIDS, or medicines such as steroid tablets or chemotherapy.  Seriously overweight (a BMI of 40 or above). | Yes / No / I prefer not to say |
| Age |  | How old are you? |  |
| Gender |  | What is your gender? | Male / Female / Other / I prefer not to say |

The UK survey also contained the brief version of the Fear of Negative Evaluation Scale (Leary, 1983), which was excluded after results showed it had no significant relation with preventive behaviors. The total sample also responded to items on trust in health care professionals, which had to be excluded from further analyses after establishing there was a flaw in the answer scale of the NL survey.

# S2 Analyses

## 2.1 Node construction

The survey items were combined to form nodes: the combination of items was either predetermined by validated scales or a fixed operationalization or based on components in the data as identified through principal component analysis (see table below). These analyses were conducted with the total sample (*n=*1022). We used principal axis factoring (PAF) with Oblimin rotation due to the expected intercorrelation between items. Extraction of components was, unless otherwise specified, based on eigenvalues greater than one. The results are discussed below. Items presented in italic were excluded from the node.

| Nodes | Approach to combining items in node |
| --- | --- |
|  |  |
| Preventive and  Repressive Behaviors | Predetermined - Operationalized with the recommended behavioral measures |
| Risk Perception | Predetermined by product of likelihood and severity |
| Health Risk | Principal Component Analysis – Single component |
| Consequences Society | Principal Component Analysis – Component identified in items on consequences |
| Consequences Economy | Principal Component Analysis – Component identified in items on consequences |
| Affect Negative | Principal Component Analysis – Component identified in items on emotions |
| Affect Positive | Principal Component Analysis – Component identified in items on emotions |
| Worries Personal | Principal Component Analysis – Component identified in items on worries |
| Worries Society | Principal Component Analysis – Component identified in items on worries |
| Vaccination Intention | Predetermined single item node |
| Measures Support | Principal Component Analysis – Component identified in items on measures |
| Measures Efficacy | Principal Component Analysis – Component identified in items on measures |
| Norm Society | Principal Component Analysis – Component identified in items on social norms |
| Norm Family Friends | Principal Component Analysis – Component identified in items on social norms |
| Control Infection | Principal Component Analysis – Single component |
| Trust Authorities | Principal Component Analysis – Single component |
| Humanitarianism | Predetermined - Validated scale |
| Need For Chaos | Predetermined - Validated scale |
| Health General | Predetermined single item node |
| Health change Physical | Predetermined single item node |
| Health change Mental | Predetermined single item node |

*Health Risk*

Kaiser-Meyer-Olkin Measure of Sampling Adequacy was .74 and Bartlett’s test of sphericity was significant, χ²(df = 6) = 1384.41, *p* < .001, supporting a rationale for performing PAF with the four items covering health risk. The items loaded on one component that explained a total of 62.9% of the variance, resulting in the node *Health Risk* with the items shown below.

| No. | Survey items | Health Risk Factor load |
| --- | --- | --- |
| 1 | Generally, I consider the health risk of becoming infected with the coronavirus to be... | .622 |
| 2 | For me personally, I consider the health risk of becoming infected with the coronavirus to be… | .662 |
| 3 | For my family and friends, I consider the health risk of becoming infected with the coronavirus to be… | .884 |
| 4 | How susceptible do you consider yourself to an infection with the coronavirus? | .679 |

*Consequences Society and Consequences Economy*

Kaiser-Meyer-Olkin Measure of Sampling Adequacy was .72 and Bartlett’s test of sphericity was significant, χ²(df = 10) = 1640.30, *p* < .001, supporting a rationale for performing PAF with the five items covering the consequences of the COVID-19 pandemic. The items loaded on two components, together explaining 73.8% of the variance, resulting in the node *Consequences Society*  (items 1 – 3) and *Consequences Economy* (items 4 and 5). Item 3 on worries about the consequences was also considered for the components on worries, but the item was included in *Consequences Society* due to a higher factor load and a larger impact on the scale reliability*.*

| No. | Survey items | Consequences Society  Factor load | Consequences Economy Factor load |
| --- | --- | --- | --- |
| 1 | I consider the societal consequences of the corona pandemic to be… | .540 |  |
| 2 | I consider the corona pandemic to be a threat that may alter society for a long time. | .848 |  |
| 3 | I worry about the consequences of the corona pandemic. | .732 |  |
| 4 | For me personally, I consider the economic consequences of the corona pandemic to be… |  | -.842 |
| 5 | For my family and friends, I consider the economic consequences of the corona pandemic to be… |  | -.799 |

*Affect Positive and Affect Negative*

Kaiser-Meyer-Olkin Measure of Sampling Adequacy was .91 and Bartlett’s test of sphericity was significant, χ²(df = 66) = 5592.85, *p* < .001, supporting a rationale for performing PAF with the 12 items covering affect. This resulted in two nodes that together explained 56.6% of the variance: *Affect Negative,* consisting of the items 1 to 9 shown below, and *Affect Positive* with items 10 and 11. The item ‘*The corona pandemic to me feels… Close to me / Far away from me’* was excluded because of its opposite (negative) factor load on the *Affect Positive* component.

| No. | Survey items | Affect Negative Factor load | Affect Positive Factor load |
| --- | --- | --- | --- |
| 1 | The corona pandemic makes me feel angry. | .676 |  |
| 2 | The corona pandemic makes me feel anxious. | .587 |  |
| 3 | The corona pandemic makes me feel sad. | .541 |  |
| 4 | The corona pandemic makes me feel confused. | .672 |  |
| 5 | The corona pandemic makes me feel overwhelmed. | .598 |  |
| 6 | The corona pandemic makes me feel frustrated. | .838 |  |
| 7 | The corona pandemic makes me feel agitated. | .843 |  |
| 8 | The corona pandemic makes me feel afraid. | .582 |  |
| 9 | The corona pandemic makes me feel out of control. | .639 |  |
| 10 | The corona pandemic makes me feel compassionate. |  | .608 |
| 11 | The corona pandemic makes me feel grateful. |  | .377 |
| *12* | *The corona pandemic to me feels… Close to me / Far away from me.* |  | -.311 |

*Worry Personal and Worry Society*

Kaiser-Meyer-Olkin Measure of Sampling Adequacy was .84 and Bartlett’s test of sphericity was significant, χ²(df = 45) = 2618.03, *p* < .001, supporting a rationale for performing PAF with 10 items covering worries. This resulted in the components *Worry Personal* (items 1 - 6) and *Worry Society* (items 7 - 9), together explaining 49.6% of the variance. The item ‘*I worry about society getting more egoistic’* was excluded due to its low factor load on both components.

| No. | Survey items | Worry Personal Factor load | Worry Virus  Factor load |
| --- | --- | --- | --- |
| 1 | I worry about getting infected with the coronavirus. | .749 |  |
| 2 | I worry about losing someone I love. | .705 |  |
| 3 | I worry about the health system being overloaded. | .603 |  |
| 4 | I worry about restricted access to food supplies. | .673 |  |
| 5 | I worry about blackouts. | .549 |  |
| 6 | I worry about becoming lonely. | .334 |  |
| 7 | I worry about schools closing. |  | .442 |
| 8 | I worry about small companies running out of business. |  | .726 |
| 9 | I worry about a recession. |  | .672 |
| *10* | *I worry about society getting more egoistic.* | *.260* | *.270* |

*Measures Support and Measures Efficacy*

Kaiser-Meyer-Olkin Measure of Sampling Adequacy was .81 and Bartlett’s test of sphericity was significant, χ²(df = 36) = 2823.68, *p* < .001, supporting a rationale for performing PAF with nine items covering attitudes toward the measures. The results initially suggested three components of which the third component had a low eigenvalue (1.040) and was difficult to interpret. We reran the reran the analysis with two fixed components, resulting in the components *Measures Support* (items 1 - 3) and *Measures Efficacy* (items 4 - 8), together explaining 53.9% of the variance. The item ‘*Social relations with other people are too important to me to consistently follow social distancing recommendations’* was excluded do to its low factor load and negative effect on the scale reliability (*a* = .64 with item and *a* = .78 without the item).

| No. | Survey items | Measures Support Factor load | Measures Efficacy Factor load |
| --- | --- | --- | --- |
| 1 | I think it is important to practice social distancing to prevent the spread of the coronavirus. | .708 |  |
| 2 | I support drastic measures imposed by authorities in my country to prevent the spread of the coronavirus (i.e. a lockdown). | .811 |  |
| 3 | As a society, we should do whatever it takes to prevent the spread of the coronavirus. | .737 |  |
| 4 | I know the recommendations from authorities in my country to prevent the spread of the coronavirus. |  | .791 |
| 5 | I follow the recommendations from authorities in my country to prevent the spread of the coronavirus. |  | .679 |
| 6 | I know how to protect myself from the coronavirus. |  | .421 |
| 7 | I think the protective behaviors will limit the spread of the coronavirus. |  | .378 |
| 8 | I feel responsible for preventing the spread of the coronavirus by displaying the protective behaviors. |  | .512 |
| *9* | *Social relations with other people are too important to me to consistently follow social distancing recommendations.* | *.256* |  |

*Norm Society and Norm Family Friends*

Kaiser-Meyer-Olkin Measure of Sampling Adequacy was .72 and Bartlett’s test of sphericity was significant, χ²(df = 15) = 1871.18, *p* < .001, supporting a rationale for performing PAF with six items covering social norms. This resulted in two components, together explaining 67.2% of the variance: *Norm Society* (items 1 and 2) and *Norm Family and Friends* (items 3 – 6).

| No. | Survey items | Norm Society Factor load | Norm Family Friends Factor load |
| --- | --- | --- | --- |
| 1 | I trust others to follow the recommendations from authorities in my country to prevent the spread of the coronavirus. | .660 |  |
| 2 | I think the majority of people display the protective behaviors. | .885 |  |
| 3 | I think my family and friends display the protective behaviors. |  | .502 |
| 4 | I see my family and friends washing their hands frequently. |  | .444 |
| 5 | My family and friends avoid social contacts. |  | .909 |
| 6 | My family and friends avoid crowded areas. |  | .851 |

*Control Infection*

Kaiser-Meyer-Olkin Measure of Sampling Adequacy was .71 and Bartlett’s test of sphericity was significant, χ²(df = 3) = 1025.26, *p* < .001, supporting a rationale for performing PAF with three items covering perceived control of getting infected. The results suggested a single component *Control Infection* with the items shown below, explaining 72.6% of the variance.

| No. | Survey items | Control Infection Factor load |
| --- | --- | --- |
| 1 | Generally, avoiding an infection with the coronavirus in the current situation is… Extremely difficult / Extremely easy | .781 |
| 2 | For me, avoiding an infection with the coronavirus in the current situation is… Extremely difficult / Extremely easy | .796 |
| 3 | For my family and friends, avoiding an infection with the coronavirus in the current situation is… Extremely difficult / Extremely easy | .726 |

*Trust Authorities*

Kaiser-Meyer-Olkin Measure of Sampling Adequacy was .93 and Bartlett’s test of sphericity was significant, χ²(df = 21) = 7088.84, *p* < .001, supporting a rationale for performing PAF with seven items covering trust in the authorities relevant for managing the pandemic. The results suggested a single component *Trust Authorities* with the items shown below, explaining 76.5% of the variance.

| No. | Survey items | Trust Authorities Factor load |
| --- | --- | --- |
| 1 | I trust the relevant authorities in my country to adequately manage the corona pandemic. | .920 |
| 2 | The relevant authorities in my country are very capable of adequately managing the corona pandemic. | .900 |
| 3 | I feel confident about the skills of the relevant authorities in my country to adequately manage the corona pandemic. | .908 |
| 4 | Sound principles seem to guide the policy of the relevant authorities in my country in relation to the corona pandemic. | .892 |
| 5 | The relevant authorities in my country try hard to be fair in dealing with the corona pandemic. | .870 |
| 6 | The relevant authorities in my country are very concerned with the welfare of people like me during the corona pandemic. | .821 |
| 7 | The needs and desires of people like me during the corona pandemic are very important to the relevant authorities in my country. | .637 |

## 2.2 Scale reliability per node

The following table contains survey statistics. It reports the number of items and scale reliability (Cronbach’s alpha or Spearman Brown coefficient in case of two items) for each node in the network consisting of more than one item for the total sample and specified for the subsamples (the UK and NL). Risk Perception excluding due to a different calculation method (product of two items instead of the mean).

| Nodes | Number of items | *a* total sample | *a* subsample UK | *a* subsample NL |
| --- | --- | --- | --- | --- |
| Preventive Behaviors | 7 | .80 | .73 | .82 |
| Repressive Behaviors* | 3 | .58 | .46 | .63 |
| Health Risk | 4 | .80 | .77 | .84 |
| Consequences Society | 3 | .75 | .70 | .79 |
| Consequences Economy | 2 | .81 | .78 | .82 |
| Affect Negative | 9 | .91 | .91 | .89 |
| Affect Positive* | 2 | .51 | .50 | .51 |
| Worry Personal | 6 | .78 | .75 | .77 |
| Worry Society* | 3 | .62 | .64 | .63 |
| Measures Support | 3 | .78 | .83 | .75 |
| Measures Efficacy | 5 | .71 | .68 | .74 |
| Norm Society | 2 | .74 | .74 | .75 |
| Norm Family Friends | 4 | .74 | .72 | .75 |
| Control Infection | 3 | .81 | .81 | .81 |
| Trust Authorities | 7 | .95 | .96 | .94 |
| Humanitarianism | 10 | .89 | .89 | .89 |
| Need For Chaos | 8 | .81 | .79 | .82 |

* The reliability of these nodes was below what one would normally prefer, but sufficient given our objective of measuring evaluations instead of designing a reliable scale (Dalege et al., 2016).

## 2.3 Information on analyses

**Classical analyses**

A Mann Whitney *U* test was conducted to compare the mean scores on the continues variables and a (two-sided) Chi-Square test for the dichotomous variables.

**Network analysis**

The network analyses were conducted with R (R Core Team, 2013) and the script is made available as supplementary material (S4). The tutorials of Dalege et al. (2017) and Haslbeck and Waldorp (2020) served as the foundation for the script. The networks were estimated with *mgm* (mixed graphical models for binary and continuous data) with k = 2 (all pairwise interactions) which was done separately for the total sample and the subsamples. Besides the values specified in the script, we used the default values. We used the package *qgraph* (Epskamp et al., 2012) to visualize the graphs, *bootnet* (Epskamp et al., 2018) for the stability and accuracy measures^[[1]](#footnote-1)^, the difference tests, and their interpretation, *NetworkComparisonTest* (van Borkulo et al., 2017) to compare the networks of the subsamples^[[2]](#footnote-2)^ and *igraph* (Csardi & Nepusz, 2006) for the community detection.

Initial community detection with the cluster walktrap algorithm resulted in rather unstable communities. We therefore conducted a community stability analysis for each sample by repeating the community analysis (1000 iterations) based on mgm and calculating how often different nodes belonged to the same community. This resulted in a score between 0 and 1, in which 0 meant that these nodes never belonged to the same community, and 1 that these nodes belonged to the same community in every iteration. The resulting matrix was visualized (see Figure S2.1 – S2.3 for the total sample and subsamples). To determine the final communities, we conducted a community detection analysis for each sample by selecting the communities with nodes that belonged to that community in over 90 percent of the iterations (see Figure S2.4 – S2.6).


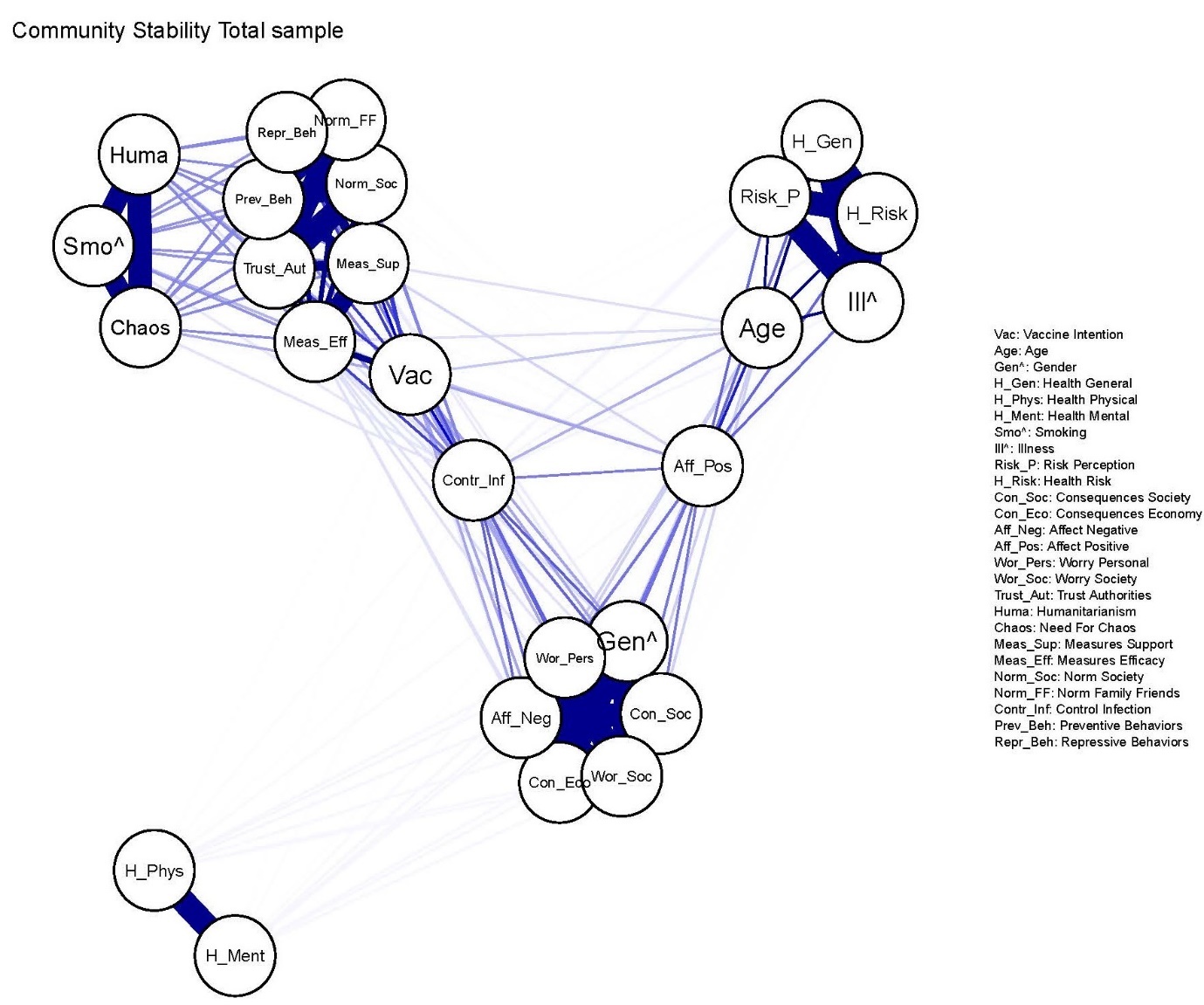


**Figure S2.1** – *Results from the community stability analysis of the total sample.*

**
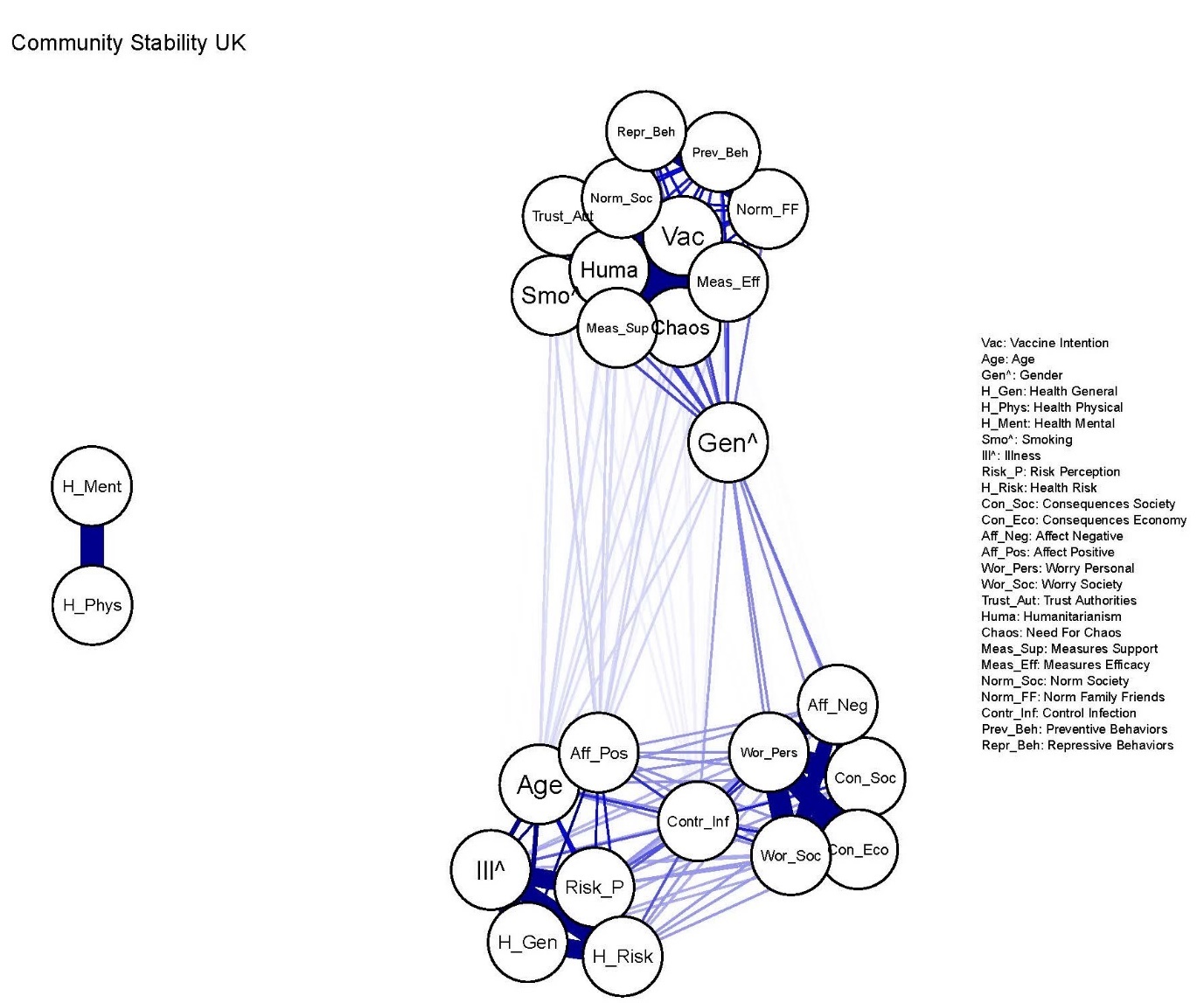
**

**Figure S2.2** – *Results from the community stability analysis of the UK subsample.*

**
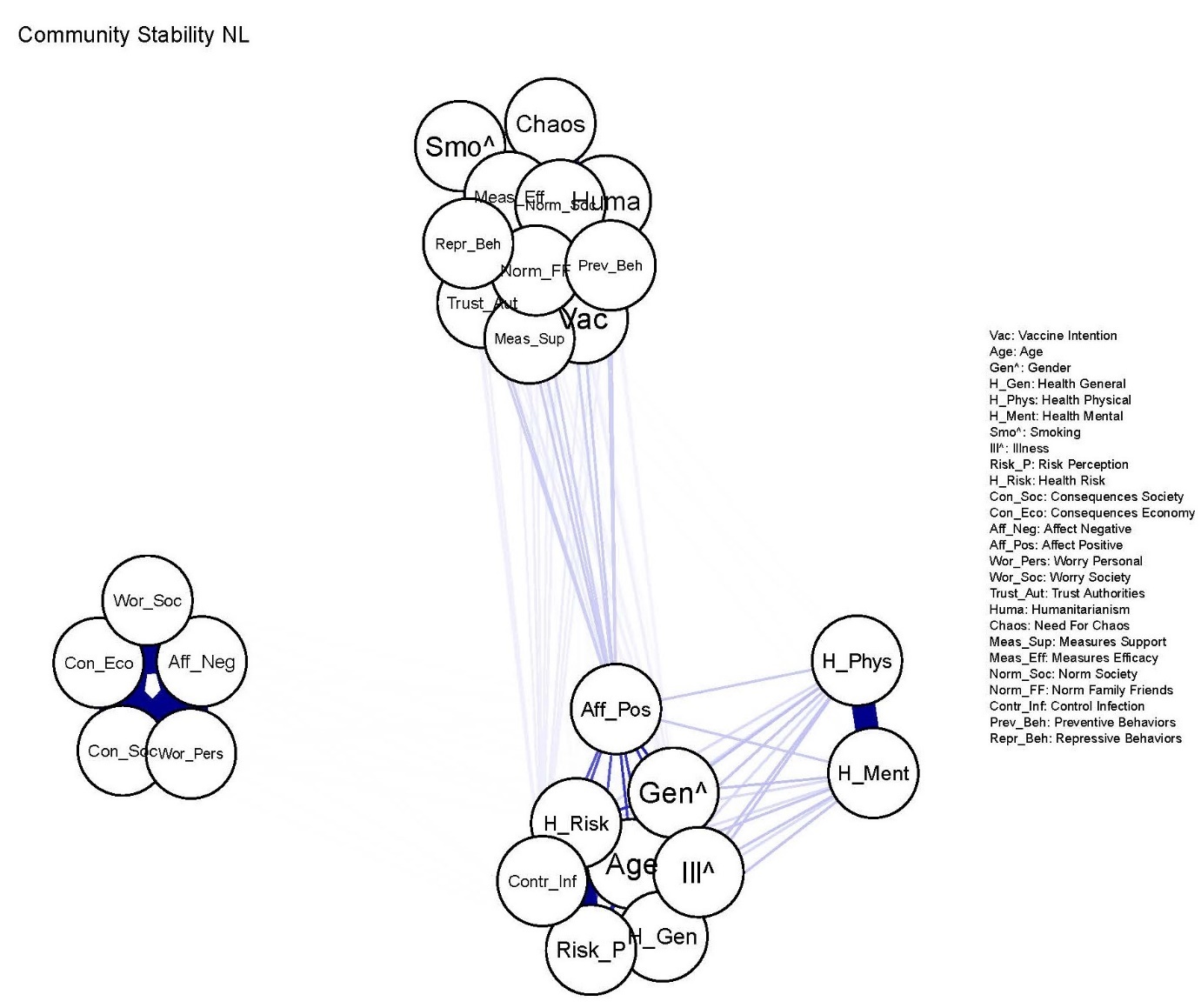
**

**Figure S2.3** – *Results from the community stability analysis of the NL subsample*.


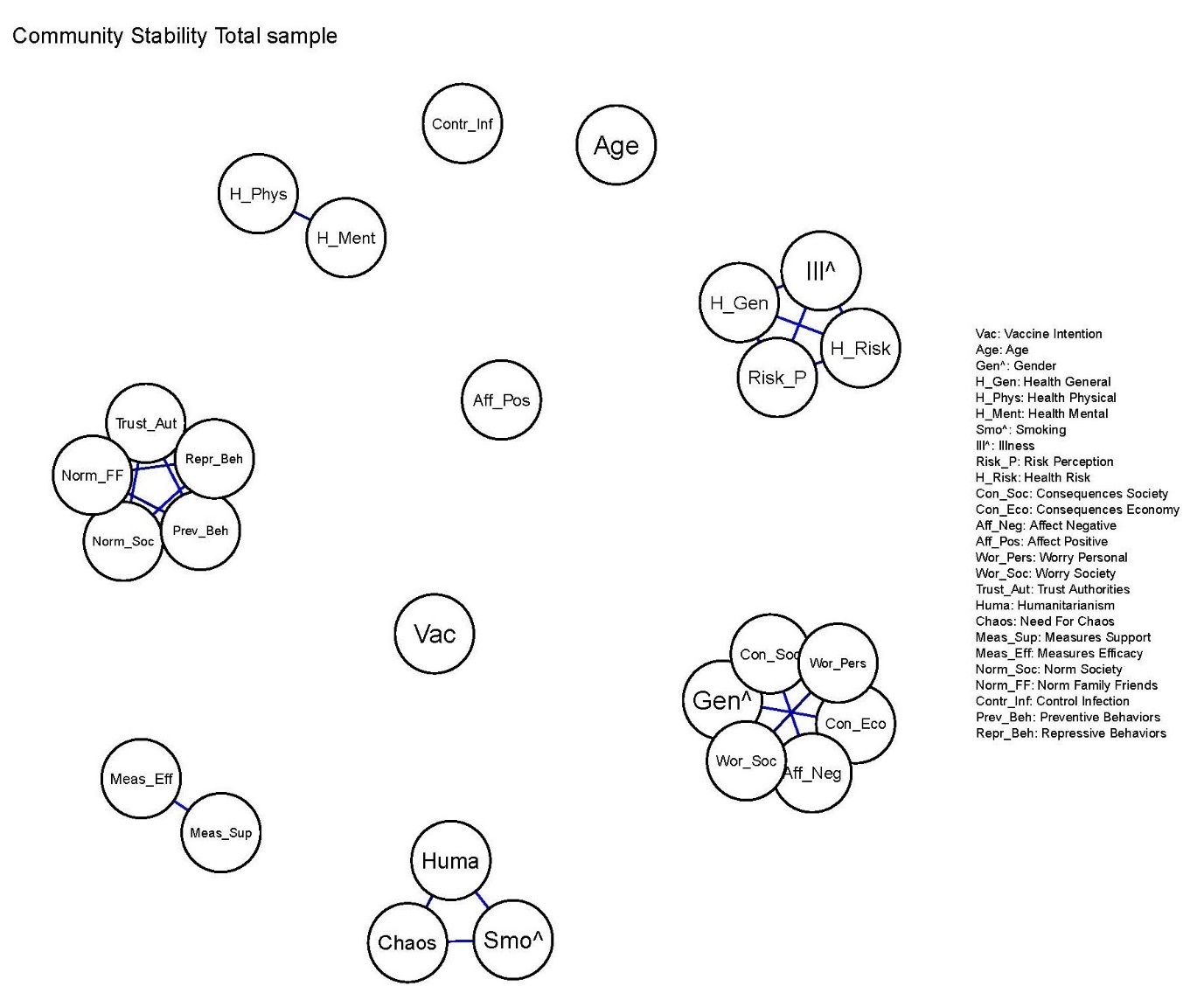


**Figure S2.4** – *Results from the community detection analysis of the total sample.*

*
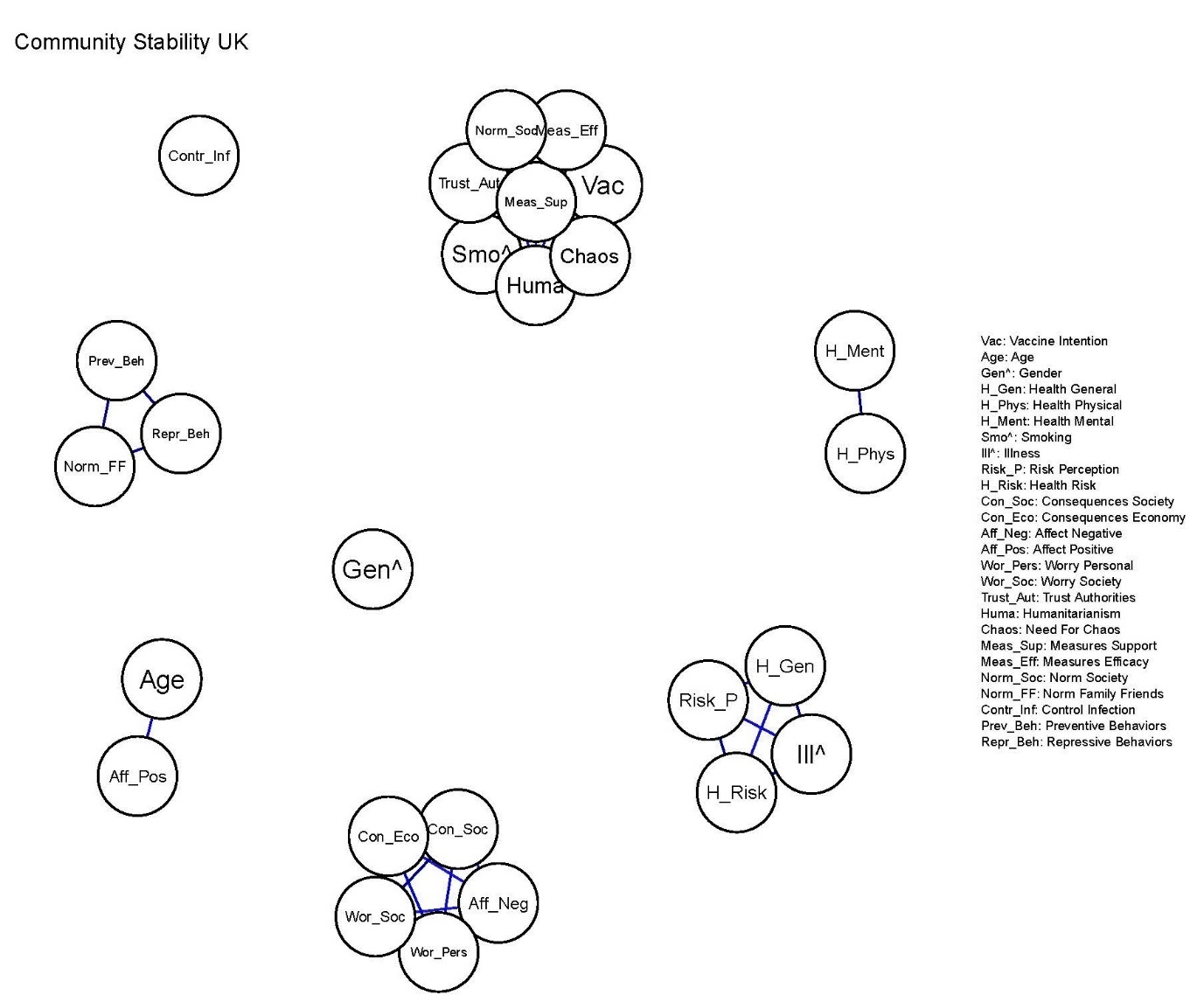
*

**Figure S2.5** – *Results from the community detection analysis of the UK subsample.*

*
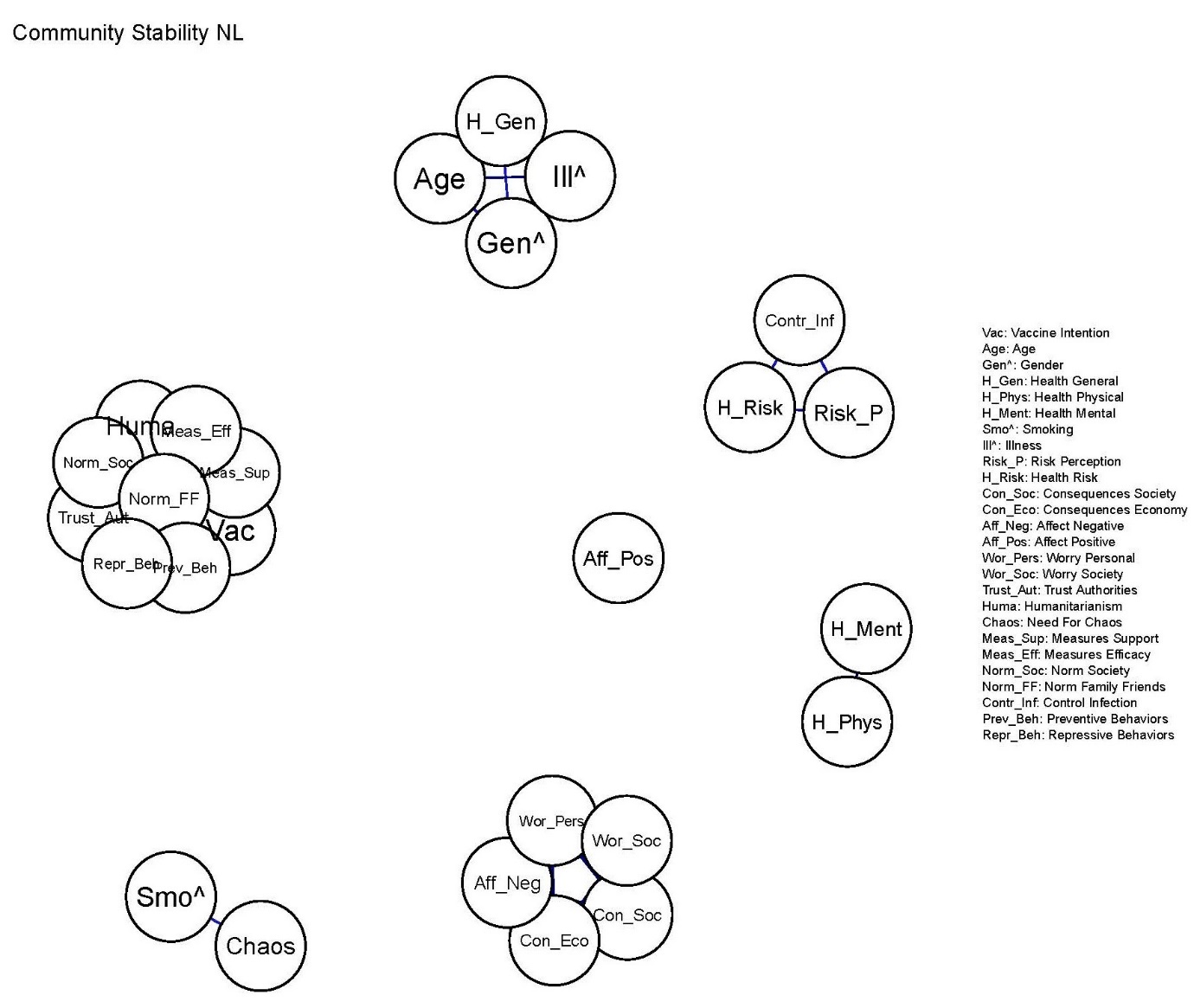
*

**Figure S2.6** – *Results from the community detection analysis of the NL subsample.*

## 2.4 Edge weights Total sample

|  | Vaccine Intention | Age | Gender | Health General | Health Physical | Health Mental | Smoking | Illness | Risk Perception | Health Risk | Consequences Society | Consequences Economy | Affect Negative | Affect Positive | Worry Personal | Worry Society | Trust Authorities | Humanitarianism | Need For Chaos | Measures Support | Measures Efficacy | Norm Society | Norm Family Friends | Control Infection | Preventive Behaviors | Repressive Behaviors |
| --- | --- | --- | --- | --- | --- | --- | --- | --- | --- | --- | --- | --- | --- | --- | --- | --- | --- | --- | --- | --- | --- | --- | --- | --- | --- | --- |
| Vaccine Intention |  |  | -.15 |  |  | -.07 |  |  |  | .11 | .06 | -.04 |  |  |  |  | .16 | -.06 | -.05 | .16 | .04 |  |  | -.05 |  |  |
| Age |  |  |  |  |  | .11 |  | .41 | .10 |  |  | -.07 |  | -.09 |  | .13 |  |  | -.10 | .09 | -.04 | .09 |  |  | .04 |  |
| Gender |  |  |  | -.09 | .14 | -.09 |  |  |  |  |  | -.07 | .18 | .15 |  |  |  |  | -.11 | .06 | .09 |  |  | -.09 | .04 | -.03 |
| Health General |  |  |  |  | .24 |  | -.08 | -.49 | -.06 | -.11 | .06 |  |  | .10 |  |  |  |  |  |  | .04 | .05 | .05 |  |  |  |
| Health Physical |  |  |  |  |  | .48 |  |  |  |  | -.04 |  | .09 |  |  |  |  |  |  |  | -.07 |  |  |  | .04 |  |
| Health Mental |  |  |  |  |  |  |  |  |  |  |  |  | -.25 | .05 |  |  |  |  |  |  |  |  |  |  |  |  |
| Smoking |  |  |  |  |  |  |  |  |  |  |  |  |  |  |  |  |  | .21 | .19 |  |  |  |  |  |  |  |
| Illness |  |  |  |  |  |  |  |  | .10 | .21 |  |  |  |  |  |  |  | -.08 |  |  |  |  | .09 | .08 |  |  |
| Risk Perception |  |  |  |  |  |  |  |  |  | .53 |  |  |  |  | .11 |  |  |  |  | -.05 |  |  |  | -.11 |  |  |
| Health Risk |  |  |  |  |  |  |  |  |  |  | .12 | .11 |  | .11 | .12 |  |  |  |  | .08 |  |  |  | -.10 |  |  |
| Consequences Society |  |  |  |  |  |  |  |  |  |  |  | .19 | .19 |  |  | .25 |  |  |  |  | .18 |  |  | -.04 |  | -.04 |
| Consequences Economy |  |  |  |  |  |  |  |  |  |  |  |  |  |  | .08 | .11 |  |  |  |  |  |  |  |  |  |  |
| Affect Negative |  |  |  |  |  |  |  |  |  |  |  |  |  | .07 | .46 | .13 |  |  |  |  | -.12 |  |  |  |  |  |
| Affect Positive |  |  |  |  |  |  |  |  |  |  |  |  |  |  |  | .08 | .05 | .18 |  | .04 | .05 |  |  |  |  | .05 |
| Worry Personal |  |  |  |  |  |  |  |  |  |  |  |  |  |  |  | .16 |  | .07 | .07 | .21 |  |  |  | -.06 | .06 | .08 |
| Worry Society |  |  |  |  |  |  |  |  |  |  |  |  |  |  |  |  | .12 |  | -.04 | -.19 |  |  |  |  |  |  |
| Trust Authorities |  |  |  |  |  |  |  |  |  |  |  |  |  |  |  |  |  |  | -.16 | .06 | .13 | .26 | -.09 |  | .07 | -.05 |
| Humanitarianism |  |  |  |  |  |  |  |  |  |  |  |  |  |  |  |  |  |  | -.03 | .10 | .11 | .15 |  |  | .04 |  |
| Need For Chaos |  |  |  |  |  |  |  |  |  |  |  |  |  |  |  |  |  |  |  |  | -.14 |  |  |  |  |  |
| Measures Support |  |  |  |  |  |  |  |  |  |  |  |  |  |  |  |  |  |  |  |  | .19 | -.09 | .10 |  | .21 | .08 |
| Measures Efficacy |  |  |  |  |  |  |  |  |  |  |  |  |  |  |  |  |  |  |  |  |  | .10 | .12 | .20 | .20 |  |
| Norm Society |  |  |  |  |  |  |  |  |  |  |  |  |  |  |  |  |  |  |  |  |  |  | .20 | .08 |  |  |
| Norm Family Friends |  |  |  |  |  |  |  |  |  |  |  |  |  |  |  |  |  |  |  |  |  |  |  |  | .27 | .06 |
| Control Infection |  |  |  |  |  |  |  |  |  |  |  |  |  |  |  |  |  |  |  |  |  |  |  |  | -.06 |  |
| Preventive Behaviors |  |  |  |  |  |  |  |  |  |  |  |  |  |  |  |  |  |  |  |  |  |  |  |  |  | .29 |
| Repressive Behaviors |  |  |  |  |  |  |  |  |  |  |  |  |  |  |  |  |  |  |  |  |  |  |  |  |  |  |

## 2.5 Edge weights UK

|  | Vaccination Intention | Age | Gender | Health General | Health Physical | Health Mental | Smoking | Illness | Risk Perception | Health Risk | Consequences Society | Consequences Economy | Affect Negative | Affect Positive | Worry Personal | Worry Society | Trust Authorities | Humanitarianism | Need For Chaos | Measures Support | Measures Efficacy | Norm Society | Norm Family Friends | Control Infection | Preventive Behaviors | Repressive Behaviors |
| --- | --- | --- | --- | --- | --- | --- | --- | --- | --- | --- | --- | --- | --- | --- | --- | --- | --- | --- | --- | --- | --- | --- | --- | --- | --- | --- |
| Vaccination Intention |  |  | -.09 |  |  |  | -.14 |  |  |  |  | -.10 |  |  |  |  | .07 |  |  | .11 | .10 | .08 |  | -.07 | -.05 |  |
| Age |  |  | .07 |  |  |  | -.12 | .30 | .20 |  |  | -.06 | -.09 | -.16 |  |  |  |  | -.16 |  |  | .13 |  | .05 |  |  |
| Gender |  |  |  |  | .13 |  | -.10 |  |  |  |  |  | .29 | .20 |  |  |  |  | -.09 | .08 | .14 | -.06 |  |  | .09 | -.13 |
| Health General |  |  |  |  | .19 |  | -.16 | -.47 | -.10 | -.09 |  |  |  | .07 |  |  |  |  |  |  | .06 | .07 | .06 |  |  |  |
| Health Physical |  |  |  |  |  | .41 |  |  |  |  |  |  | .05 |  |  |  |  |  |  |  |  |  |  |  |  |  |
| Health Mental |  |  |  |  |  |  |  |  |  |  |  |  | -.29 |  |  |  |  |  |  |  |  |  |  |  |  |  |
| Smoking |  |  |  |  |  |  |  | -.16 |  |  |  |  |  |  | .12 |  | .10 | .35 | .27 |  |  | .11 |  |  |  |  |
| Illness |  |  |  |  |  |  |  |  |  | .20 |  |  |  |  | .06 |  |  |  |  | -.14 |  |  | .08 | .10 |  |  |
| Risk Perception |  |  |  |  |  |  |  |  |  | .52 |  |  | .06 |  | .07 |  |  |  |  |  |  |  |  |  |  |  |
| Health Risk |  |  |  |  |  |  |  |  |  |  | .13 | .15 |  | .10 | .20 | -.14 |  |  |  | .10 |  |  |  | -.07 |  |  |
| Consequences Society |  |  |  |  |  |  |  |  |  |  |  | .21 | .23 |  |  | .23 |  |  |  |  | .21 |  |  |  |  | -.08 |
| Consequences Economy |  |  |  |  |  |  |  |  |  |  |  |  |  | .05 |  | .16 |  |  |  |  | -.05 |  |  |  |  |  |
| Affect Negative |  |  |  |  |  |  |  |  |  |  |  |  |  |  | .39 | .08 |  |  |  |  | -.18 |  |  |  |  |  |
| Affect Positive |  |  |  |  |  |  |  |  |  |  |  |  |  |  |  | .06 |  | .21 |  |  | .09 | .05 |  |  |  |  |
| Worry Personal |  |  |  |  |  |  |  |  |  |  |  |  |  |  |  | .22 |  |  |  | .17 |  |  | -.06 | -.08 | .07 |  |
| Worry Society |  |  |  |  |  |  |  |  |  |  |  |  |  |  |  |  | .08 | .06 | -.09 | -.11 |  |  |  |  |  |  |
| Trust Authorities |  |  |  |  |  |  |  |  |  |  |  |  |  |  |  |  |  | -.06 | -.14 | .11 | .09 | .29 | -.12 |  | .08 | -.06 |
| Humanitarianism |  |  |  |  |  |  |  |  |  |  |  |  |  |  |  |  |  |  | -.09 | .11 | .07 | .09 |  |  |  |  |
| Need For Chaos |  |  |  |  |  |  |  |  |  |  |  |  |  |  |  |  |  |  |  | -.08 | -.13 | -.06 |  |  |  |  |
| Measures Support |  |  |  |  |  |  |  |  |  |  |  |  |  |  |  |  |  |  |  |  | .17 | -.11 | .09 |  | .21 |  |
| Measures Efficacy |  |  |  |  |  |  |  |  |  |  |  |  |  |  |  |  |  |  |  |  |  |  | .10 | .21 | .16 | .07 |
| Norm Society |  |  |  |  |  |  |  |  |  |  |  |  |  |  |  |  |  |  |  |  |  |  | .24 |  |  |  |
| Norm Family Friends |  |  |  |  |  |  |  |  |  |  |  |  |  |  |  |  |  |  |  |  |  |  |  |  | .30 | .07 |
| Control Infection |  |  |  |  |  |  |  |  |  |  |  |  |  |  |  |  |  |  |  |  |  |  |  |  |  |  |
| Preventive Behaviors |  |  |  |  |  |  |  |  |  |  |  |  |  |  |  |  |  |  |  |  |  |  |  |  |  | .30 |
| Repressive Behaviors |  |  |  |  |  |  |  |  |  |  |  |  |  |  |  |  |  |  |  |  |  |  |  |  |  |  |

## 2.6 Edge weights NL

|  | Vaccination Intention | Age | Gender | Health General | Health Physical | Health Mental | Smoking | Illness | Risk Perception | Health Risk | Consequences Society | Consequences Economy | Affect Negative | Affect Positive | Worry Personal | Worry Society | Trust Authorities | Humanitarianism | Need For Chaos | Measures Support | Measures Efficacy | Norm Society | Norm Family Friends | Control Infection | Preventive Behaviors | Repressive Behaviors |
| --- | --- | --- | --- | --- | --- | --- | --- | --- | --- | --- | --- | --- | --- | --- | --- | --- | --- | --- | --- | --- | --- | --- | --- | --- | --- | --- |
| Vaccination Intention |  | .05 | -.13 |  |  |  |  |  |  | .11 | .09 |  |  | -.07 |  |  | .25 | -.07 | -.04 | .15 |  |  |  |  | .11 |  |
| Age |  |  | -.05 |  |  | .06 |  | .42 |  |  |  |  |  |  |  | .12 | -.09 |  |  | .16 |  |  |  |  |  |  |
| Gender |  |  |  | -.21 | .13 | -.10 |  |  | -.08 |  |  |  | .10 | .09 |  |  |  |  | -.07 | .11 |  |  |  | -.13 |  |  |
| Health General |  |  |  |  | .26 |  |  | -.48 |  | -.11 | .05 |  |  | .08 |  | .07 | .07 |  |  |  |  |  |  |  |  |  |
| Health Physical |  |  |  |  |  | .50 |  |  |  |  |  |  | .08 |  |  |  |  |  |  |  | -.07 |  |  |  |  |  |
| Health Mental |  |  |  |  |  |  |  |  |  |  |  |  | -.16 | .09 |  |  |  |  |  | .08 |  | -.05 |  |  |  |  |
| Smoking |  |  |  |  |  |  |  |  |  |  |  |  |  |  |  |  |  | .16 | .13 |  |  |  |  |  |  |  |
| Illness |  |  |  |  |  |  |  |  | .15 | .16 |  |  |  |  |  |  |  | -.20 |  |  |  |  | .07 |  |  |  |
| Risk Perception |  |  |  |  |  |  |  |  |  | .50 |  |  |  |  | .15 |  |  |  |  |  |  |  |  | -.15 |  |  |
| Health Risk |  |  |  |  |  |  |  |  |  |  |  | .11 |  | .08 | .13 |  |  |  |  | .10 |  |  |  | -.10 |  |  |
| Consequences Society |  |  |  |  |  |  |  |  |  |  |  | .18 | .17 |  | .08 | .21 | -.04 | .04 | -.08 |  | .15 |  |  |  |  |  |
| Consequences Economy |  |  |  |  |  |  |  |  |  |  |  |  |  |  |  | .12 |  |  |  |  |  |  |  |  |  |  |
| Affect Negative |  |  |  |  |  |  |  |  |  |  |  |  |  | .13 | .44 | .18 |  |  |  |  | -.07 |  |  |  |  |  |
| Affect Positive |  |  |  |  |  |  |  |  |  |  |  |  |  |  |  |  | .15 | .12 |  |  |  |  |  |  |  | .06 |
| Worry Personal |  |  |  |  |  |  |  |  |  |  |  |  |  |  |  | .18 |  | .11 |  | .11 | -.11 |  |  |  |  | .07 |
| Worry Society |  |  |  |  |  |  |  |  |  |  |  |  |  |  |  |  |  |  |  | -.14 |  |  |  |  |  |  |
| Trust Authorities |  |  |  |  |  |  |  |  |  |  |  |  |  |  |  |  |  | .08 | -.13 | .08 | .18 | .17 |  |  |  |  |
| Humanitarianism |  |  |  |  |  |  |  |  |  |  |  |  |  |  |  |  |  |  |  |  | .12 | .17 |  |  | .07 |  |
| Need For Chaos |  |  |  |  |  |  |  |  |  |  |  |  |  |  |  |  |  |  |  | .04 | -.14 |  |  | .04 |  |  |
| Measures Support |  |  |  |  |  |  |  |  |  |  |  |  |  |  |  |  |  |  |  |  | .17 |  | .06 |  | .18 | .08 |
| Measures Efficacy |  |  |  |  |  |  |  |  |  |  |  |  |  |  |  |  |  |  |  |  |  | .14 | .13 | .13 | .21 |  |
| Norm Society |  |  |  |  |  |  |  |  |  |  |  |  |  |  |  |  |  |  |  |  |  |  | .14 | .14 |  |  |
| Norm Family Friends |  |  |  |  |  |  |  |  |  |  |  |  |  |  |  |  |  |  |  |  |  |  |  |  | .23 |  |
| Control Infection |  |  |  |  |  |  |  |  |  |  |  |  |  |  |  |  |  |  |  |  |  |  |  |  |  |  |
| Preventive Behaviors |  |  |  |  |  |  |  |  |  |  |  |  |  |  |  |  |  |  |  |  |  |  |  |  |  | .27 |
| Repressive Behaviors |  |  |  |  |  |  |  |  |  |  |  |  |  |  |  |  |  |  |  |  |  |  |  |  |  |  |

## 2.7 NCT weights (UK – NL)

|  | Vaccination Intention | Age | Gender | Health General | Health Physical | Health Mental | Smoking | Illness | Risk Perception | Health Risk | Consequences Society | Consequences Economy | Affect Negative | Affect Positive | Worry Personal | Worry Society | Trust Authorities | Humanitarianism | Need For Chaos | Measures Support | Measures Efficacy | Norm Society | Norm Family Friends | Control Infection | Preventive Behaviors | Repressive Behaviors |
| --- | --- | --- | --- | --- | --- | --- | --- | --- | --- | --- | --- | --- | --- | --- | --- | --- | --- | --- | --- | --- | --- | --- | --- | --- | --- | --- |
| Vaccination Intention |  |  |  |  |  |  |  |  |  |  |  | -0,10 |  |  |  |  | -0,18 |  |  |  |  |  |  |  | -0,17 |  |
| Age |  |  |  |  |  |  |  |  | 0,20 |  |  |  | -0,09 | -0,16 |  |  |  |  | -0,16 | -0,16 |  |  |  |  |  |  |
| Gender |  |  |  |  |  |  |  |  |  |  |  |  | 0,19 | 0,11 |  |  |  |  |  |  |  |  |  |  | 0,09 |  |
| Health General |  |  |  |  |  |  |  |  |  |  |  |  |  |  |  |  |  |  |  |  | 0,06 |  |  |  |  |  |
| Health Physical |  |  |  |  |  |  |  |  |  |  |  |  |  |  |  |  |  |  |  |  |  |  |  |  |  |  |
| Health Mental |  |  |  |  |  |  |  |  |  |  |  |  | -0,12 |  |  |  |  |  |  | -0,08 |  |  |  |  |  |  |
| Smoking |  |  |  |  |  |  |  |  |  |  |  |  |  |  |  |  |  |  |  |  |  |  |  |  |  |  |
| Illness |  |  |  |  |  |  |  |  |  |  |  |  |  |  |  |  |  |  |  |  |  |  |  |  |  |  |
| Risk Perception |  |  |  |  |  |  |  |  |  |  |  |  |  |  |  |  |  |  |  |  |  |  |  |  |  |  |
| Health Risk |  |  |  |  |  |  |  |  |  |  |  |  |  |  |  |  |  |  |  |  |  |  |  |  |  |  |
| Consequences Society |  |  |  |  |  |  |  |  |  |  |  |  |  |  |  |  |  |  |  |  |  |  |  |  |  |  |
| Consequences Economy |  |  |  |  |  |  |  |  |  |  |  |  |  |  |  |  |  |  |  |  |  |  |  |  |  |  |
| Affect Negative |  |  |  |  |  |  |  |  |  |  |  |  |  |  |  |  |  |  |  |  |  |  |  |  |  |  |
| Affect Positive |  |  |  |  |  |  |  |  |  |  |  |  |  |  |  |  | -0,15 |  |  |  |  |  |  |  |  |  |
| Worry Personal |  |  |  |  |  |  |  |  |  |  |  |  |  |  |  |  |  |  |  |  |  |  |  |  |  |  |
| Worry Society |  |  |  |  |  |  |  |  |  |  |  |  |  |  |  |  |  |  |  |  |  |  |  |  |  |  |
| Trust Authorities |  |  |  |  |  |  |  |  |  |  |  |  |  |  |  |  |  | -0,14 |  |  | -0,09 |  |  |  |  |  |
| Humanitarianism |  |  |  |  |  |  |  |  |  |  |  |  |  |  |  |  |  |  |  |  |  |  |  |  |  |  |
| Need For Chaos |  |  |  |  |  |  |  |  |  |  |  |  |  |  |  |  |  |  |  | -0,12 |  |  |  |  |  |  |
| Measures Support |  |  |  |  |  |  |  |  |  |  |  |  |  |  |  |  |  |  |  |  |  |  |  |  |  |  |
| Measures Efficacy |  |  |  |  |  |  |  |  |  |  |  |  |  |  |  |  |  |  |  |  |  |  |  |  |  |  |
| Norm Society |  |  |  |  |  |  |  |  |  |  |  |  |  |  |  |  |  |  |  |  |  |  |  |  |  |  |
| Norm Family Friends |  |  |  |  |  |  |  |  |  |  |  |  |  |  |  |  |  |  |  |  |  |  |  |  |  |  |
| Control Infection |  |  |  |  |  |  |  |  |  |  |  |  |  |  |  |  |  |  |  |  |  |  |  |  |  |  |
| Preventive Behaviors |  |  |  |  |  |  |  |  |  |  |  |  |  |  |  |  |  |  |  |  |  |  |  |  |  |  |
| Repressive Behaviors |  |  |  |  |  |  |  |  |  |  |  |  |  |  |  |  |  |  |  |  |  |  |  |  |  |  |

# S3 COVID-19 Timeline UK NL

## 3.1 Visual timeline


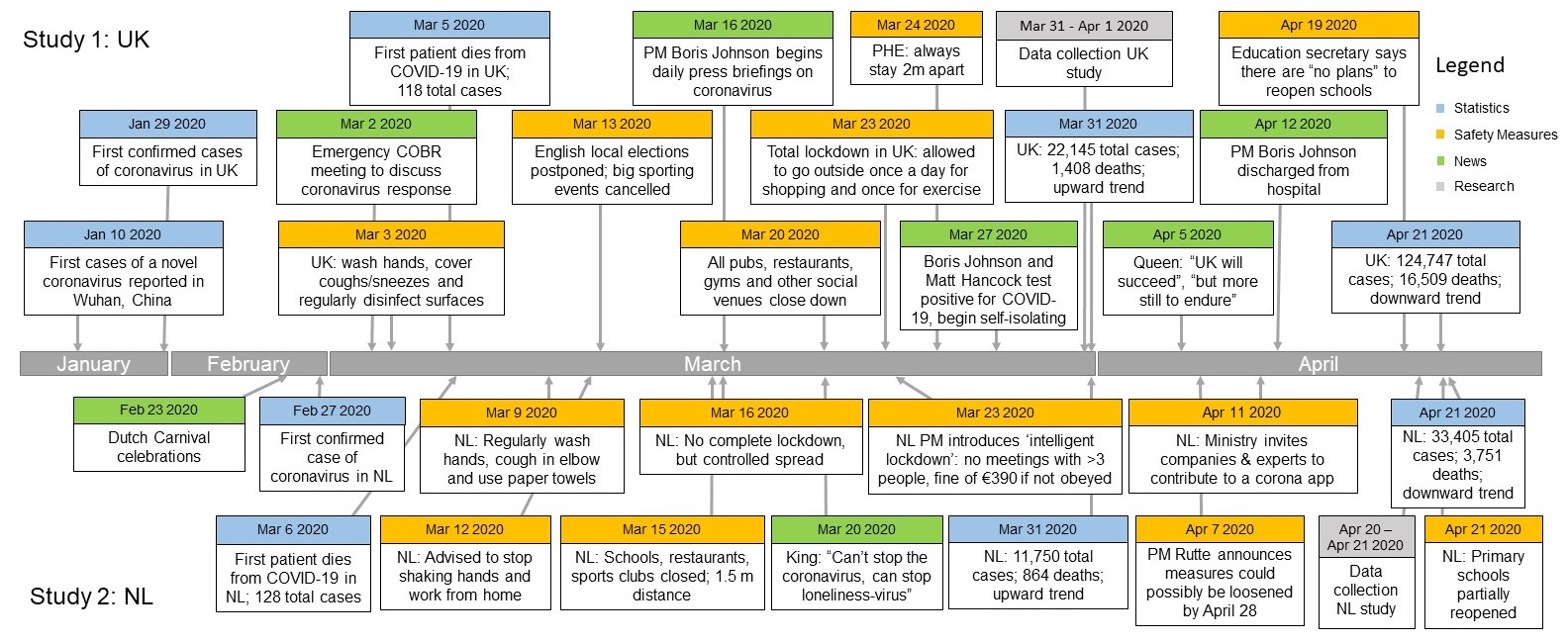


## 3.2 Data The United Kingdom

| **Date** | **UK/inter-national** | **Theme^[[3]](#footnote-3)^** | **Description of the event** |
| --- | --- | --- | --- |
| 23-6-2016 | UK | Context | The citizens of the UK vote to exit the EU with a majority 51,9 percent of the votes. Prime Minister David Cameron announces his resignation, saying the country deserves a leader committed to carrying out the will of the people. |
| 13-7-2016 | UK | Context | Theresa May, leader of the conservative party, becomes the UK's new Prime Minister. Boris Johnson is appointed as the new foreign secretary. |
| 19-4-2017 | UK | Context | After announcing a snap general election on 18-04-2017, Theresa May and the conservatives lose their majority in the parliament. The election was called to try to strengthen the conservatives position in the talks with the EU on Brexit. |
| 29-3-2017 | UK | Context | The UK triggers article 50 of the Lisbon treaty, the official mechanism that makes leaving the EU possible. The Brexit negotiations between the British parliament and the EU have officially started and are planned to last until the 29th of March 2019. |
| 30-3-2017 | UK | Context | Scotland's first minister has signed a formal letter to ask for permission to hold a second Scottish Independence referendum to detach itself from the UK. The Scottish government wants the Scottish people to have a choice over their future in wake of the results of the Brexit vote, where over 60 percent of Scottish people voted to remain in the EU. The first Scottish Independence referendum was held in 2014 and a majority of 55% voted to remain part of the UK. During the first vote, some parties argued that remaining part of the UK was the only way for Scotland to remain part of the EU. |
| 24-7-2019 | UK | Context | Boris Johnson, leader of the conservative party, replaces Theresa May as the UK's new prime minister with the promise of getting Brexit done by the 31st of October. |
| 12-12-2019 | UK | Context | The Conservatives win the 2019 United Kingdom general election, with a landslide majority of 80 seats in the parliament. Boris Johnson remains prime minister, beating his opponent from the Labour party Jeremy Corbyn. |
| 31-12-2019 | International | Statistics | WHO is informed of cases of pneumonia of unknown cause detected in the city of Wuhan, in the Hubei Province, China. |
| 10-1-2020 | International | Statistics | The WHO reports that a novel coronavirus has emerged in China. After the first reports of pneumonia came in China came in, it has been confirmed by Chinese authorities that this is caused by a novel coronavirus. |
| 22-1-2020 | UK | Safety measures | Public Health England increases the risk levels for the novel coronavirus from 'very low' to ' low'. Health teams will be monitoring passengers from Wuhan and the Hubei region. |
| 28-1-2020 | UK | Safety measures | The Foreign and Commonwealth office recommends people not to travel to the Hubei region and alarms people to be cautious for the virus when travelling to China. People who have returned from Wuhan have been recommended to stay inside and avoid contact with other people as much as possible. |
| 29-1-2020 | UK | Statistics | The first two cases of the coronavirus in the UK have been confirmed. The people have a Chinese nationality and stayed in a hotel on holiday. |
| 30-1-2020 | International | Safety measures | The WHO declares the coronavirus outbreak as a global emergency, demanding temporary safety measures to be made in all countries. Countries have to prove their healthcare safety measures are based on sound scientific reasoning. According to the WHO, declaring an international emergency helps strengthen global coordination, transparency and increases support for countries with less developed healthcare systems. |
| 31-1-2020 | International | Statistics | First case of coronavirus confirmed in Spain. |
| 6-2-2020 | UK | Statistics | A third person in the UK has tested positive for the coronavirus. He picked up the virus on a business conference in Singapore and he has later been linked with at least 11 other cases, making him a so-called "super-spreader". |
| 7-2-2020 | International | News | Dr Wenliang, a Chinese doctor who tried to warn people about the possible spread of the virus, died from the coronavirus. |
| 9-2-2020 | UK | Statistics | The total number of confirmed cases is 4. |
| 10-2-2020 | UK | Safety measures | Government announces strengthened legal powers to deal with the coronavirus. This ensures that the NHS staff has enough power to keep people in isolation for longer than 14 days if they believe there is a valid reason to take such actions. |
| 11-2-2020 | International | News | The WHO announces official names for the novel coronavirus disease (COVID-19) and the virus that causes it (SARS-CoV-2). |
| 23-2-2020 | UK | Statistics | Since yesterday, the total amount of patients that have tested positive has grown with 4. This brings the total number of cases to 13. |
| 28-2-2020 | UK | Statistics | The first British victim dies of COVID-19 onboard the Diamond Princess, a cruise ship stationed near the coast of Japan. |
| 1-3-2020 | UK | Statistics | Since yesterday, the total amount of patients that have tested positive has grown with 13. This brings the total number of cases to 36. |
| 2-3-2020 | UK | News | PM Boris Johnson will lead a meeting of the government emergency COBR committee to discuss countermeasures to fight off the coronavirus outbreak. |
| 2-3-2020 | UK | News | In an interview with the BBC, Prime Minister Boris Johnson warns the public that there could be a very significant expansion in the number of cases of coronavirus in the UK. |
| 3-3-2020 | UK | Safety measures | The Government announces a new action plan. The plan describes measures such as closing some schools, encouraging homeworking, reducing the number of large-scale gatherings and creating more publicity for the promotion of good hygiene. They advise to stay at home if you show symptoms, wash hands more frequently, cough or sneeze in a tissue or your elbow/sleeve and to disinfect regularly touched objects and surfaces. |
| 4-3-2020 | UK | Statistics | The UK reported the largest one-day increase in cases of the virus so far, as 38 new cases bring the total to 89. |
| 4-3-2020 | International | Safety measures | Italy announces it is closing down schools and universities nationwide until mid-march. |
| 5-3-2020 | UK | Statistics | First reported coronavirus fatality in the UK. The woman was in her 70s and had not been in contact with people that had been confirmed with the coronavirus. Total amount of cases grows to: 118. |
| 8-3-2020 | UK | Statistics | Since yesterday, the total amount of patients that have tested positive has grown with 67. This brings the total number of cases to 277 and a total of 2 patients have died. |
| 9-3-2020 | International | Safety measures | Italy's prime minister Guiseppe Conte announces a nationwide forced quarantine on national television. Only people with a valid work or family reason are allowed to travel. All social events are cancelled. |
| 10-3-2020 | UK | Statistics | Since yesterday, the total amount of patients that have tested positive has grown with 50 and 3 patients have died. This brings the total number of cases to 373 and the amount of patients that died to 6. |
| 10-3-2020 | UK | News | Nadine Dorries, a junior health minister, becomes the first member of parliament to test positive for COVID-19. |
| 11-3-2020 | International | News | The WHO declares that COVID-19 can be characterized as a pandemic. This does not change the WHO's assessment of the threat posed by the virus, neither does it change what the WHO's actions are towards solving the situation. According to the WHO, the characterization as a pandemic serves to warn countries and to call for countries to 'take urgent and aggressive action'. |
| 13-3-2020 | UK | Safety measures | English local elections are postponed for a year due to coronavirus. |
| 13-3-2020 | UK | Safety measures | Big sporting events like the London Marathon and all premier league football games are postponed. |
| 14-3-2020 | International | Safety measures | The Spanish government has formally declared a state of emergency over the coronavirus, placing the country in lockdown for the next weeks. |
| 15-3-2020 | UK | Statistics | Since yesterday, the total amount of patients that have tested positive has grown with 251 and 14 patients have died. This brings the total number of cases to 1.395 and the amount of patients that died to 35. |
| 15-3-2020 | UK | Safety measures | Matt Hancock, secretary of state for health and social care, announces in an interview with the BBC that citizens over the age of 70 would be told to self-isolate within the coming weeks. |
| 16-3-2020 | UK | News | The UK government has been facing backlash from scientists and the public because of the lack of safety measures against the coronavirus. The government admits that it has had problems communicating a clear message on the communication of the precautions. |
| 16-3-2020 | UK | News | Prime minister Boris Johnson begins daily press briefings intended to focus on informing the public on how to protect themselves from the coronavirus. The briefings also routinely feature advice from professor Chris Whitty, the government's chief medical advisor and head of the public health profession, and sir Patrick Vallance, chief scientific advisor and head of the Government Science and Engineering. |
| 18-3-2020 | UK | Safety measures | The government launches its first TV-advert featuring Chief Medical Officer Chris Witty. The ad informs people about the symptoms of COVID-19 and advices people with symptoms to stay at home. The slogan is "Protect yourself, protect others, protect the NHS". |
| 20-3-2020 | UK | Safety measures | The UK government orders all pubs, restaurants, gyms and other social venues to close due to the coronavirus. The announcement was made by Boris Johnson in a news conference. |
| 22-3-2020 | UK | Statistics | The WHO reported no daily growth statistics for March 22. The total number of cases is 5.018 and the amount of patients that died is 233. |
| 23-3-2020 | UK | Safety measures | Prime Minister Boris Johnson announces a total lockdown to the people in the UK in a speech on national television. People are advised to only go outside to buy basic necessities, exercise once a day, or to go to work if you have an essential job. These rules are backed by police enforcement and people who fail comply with the rules will face police fines. People who choose to ignore the government's advice on social distancing described as very 'selfish' by Health Secretary Matt Hancock. |
| 24-3-2020 | UK | Safety measures | The PHE shares a new update, telling people to always stay 2 meters apart. |
| 24-3-2020 | UK | News | Photographs of overcrowded subways showed up on different media platforms. Mayor of London Khan told people not to stop all non-essential use of public transport, after the city cut subway services by 50%. |
| 25-3-2020 | UK | Safety measures | The British government launches an official ad campaign, with the central message being: "Stay home, Protect the NHS, Save Lives". The adverts will be shown across all types of traditional, digital and social media. The tv-advert again features Chief Medical Officer Chris Witty, saying people should only go out if absolutely necessary, always stay 2 meters apart and not meet others outside your household. |
| 27-3-2020 | UK | News | Thousands of people in the UK clap from their homes in support the NHS staff, because of an initiative called "Clap for our carers". |
| 27-3-2020 | UK | News | Prime Minister Boris Johnson and Health Secretary Matt Hancock test positive for COVID-19 and stated that they have started to self-isolate. |
| 29-3-2020 | UK | Statistics | Since yesterday, the total amount of patients that have tested positive has grown with 2433 and 260 patients have died. This brings the total number of cases to 19.526 and the amount of patients that died to 1.019. |
| 30-3-2020 | UK | News | Specialized units of the UK government are being set up to combat the spread of misinformation on social media regarding the coronavirus. The Government also relaunches an old campaign against the spreading of fake news with the slogan: "Don't feed the beast', urging the public to think carefully about what they share on social media. |
| 31-3-2020 | UK | Statistics | Since yesterday, the total amount of patients that have tested positive has grown with 2.619 and 180 patients have died. This brings the total number of cases to 22.145 and the amount of patients that died to 1.408. |
| 31-3-2020 | UK | Research | Start of data collection of the present study. |
| 1-4-2020 | UK | News | The government launches a new ad campaign with more serious warning messages, like "if you go out, you can spread it. People will die". The older message "Stay home, Protect the NHS, Save lives" is still shown on these adverts. These messages are being shown on newspapers and social media. |
| 1-4-2020 | UK | Safety measures | The UK government announces it will massively increase coronavirus testing, as the UK saw its biggest increase in deaths yet'. The government faced pressure to do more tests on the NHS staff, because, at this point in time, only 2,000 frontline workers had been tested. |
| 1-4-2020 | UK | Research | End of data collection. |
| 2-4-2020 | UK | News | For the second time, people across the UK have taken part in the "Clap for Carers" initiative, applauding the NHS staff and other key workers. The event is now expected to happen every Thursday. |
| 5-4-2020 | UK | Statistics | Since yesterday, the total amount of patients that have tested positive has grown with 3.735 and 708 patients have died. This brings the total number of cases to 41.907 and the amount of patients that died to 4.313. |
| 5-4-2020 | UK | News | Queen Elizabeth II addresses the nation, thanking people for following the social distancing rules and praising essential workers. She also told the UK citizens that the UK 'will succeed' in the fight against the coronavirus, but that ' they may have more still to endure'. |
| 5-4-2020 | UK | News | Prime Minister Boris Johnson is admitted to St Thomas Hospital in London as a precautionary measure after his coronavirus symptoms persist. |
| 7-4-2020 | UK | News | Prime Minister Boris Johnson has been moved to the intensive care in the hospital, after his symptoms worsened. |
| 12-4-2020 | UK | Statistics | Since yesterday, the total amount of patients that have tested positive has grown with 8.719 and 917 patients have died. This brings the total number of cases to 78.995 and the amount of patients that died to 9.875. |
| 12-4-2020 | UK | News | Prime Minister Boris Johnson leaves the hospital and continues his recovery from home. In a message to the public, he thanks the NHS for saving his life and says the UK is “making progress in this incredible national battle against coronavirus”. |
| 14-4-2020 | UK | News | Arsonists attack phone masts because of conspiracy theories that 5G-network causes spreading of the coronavirus. |
| 16-4-2020 | UK | Safety measures | In a televised briefing, foreign secretary Dominic Raab, deputizing for Boris Johnson, announces that measures are extended for 'at least' three more weeks. |
| 19-4-2020 | UK | News | Multiple media outlets report of a shortage in personal protective equipment (PPE), like face masks and gloves, in hospitals around the country. |
| 19-4-2020 | UK | News | Education secretary Gavin Williamson announces: "There are currently no plans to have schools open over the summer period". |
| 20-4-2020 | UK | News | The UK Government launches a support package where employers can apply for a furlough payment due to the coronavirus. 140.000 UK companies apply for the furlough scheme on the first day. |
| 21-4-2020 | UK | Statistics | Since yesterday, the total amount of patients that have tested positive has grown with 4.676 and 449 patients have died. This brings the total number of cases to 124.747 and the amount of patients that died to 16.509. |
| 21-4-2020 | International | Research | Start of data collection of the NL-study. |
| 23-4-2020 | International | Research | End of data collection of the NL-study. |

## 3.3 Data The Netherlands

| **Date** | **NL/inter-national** | **Theme^[[4]](#footnote-4)^** | **Description of the event** |
| --- | --- | --- | --- |
| 31-12-2019 | International | Statistics | WHO is informed of cases of pneumonia of unknown cause detected in the city of Wuhan, in the Hubei Province, China. |
| 10-1-2020 | International | Statistics | The WHO reports that a novel coronavirus has emerged in China. After the first reports of pneumonia came in China came in, it has been confirmed by Chinese authorities that this is caused by a novel coronavirus. |
| 30-1-2020 | International | Safety measures | The WHO declares the coronavirus outbreak as a global emergency, demanding temporary safety measures to be made in all countries. Countries have to prove their healthcare safety measures are based on sound scientific reasoning. According to the WHO, declaring an international emergency helps strengthen global coordination, transparency and increases support for countries with less developed healthcare systems. |
| 31-1-2020 | International | Statistics | First case of coronavirus confirmed in Spain. |
| 2-2-2020 | NL | News | Flight with over 15 Dutch people returns from Wuhan after waiting several days to be able to return. |
| 7-2-2020 | International | News | Dr Wenliang, a Chinese doctor who tried to warn people about the possible spread of the virus, died from the coronavirus. |
| 11-2-2020 | International | News | The WHO announces official names for the novel coronavirus disease (COVID-19) and the virus that causes it (SARS-CoV-2). |
| 23-2-2020 | NL | Safety measures | After the virus starts to spread in other European countries like Italy, the Dutch government is not yet implementing extra safety measures in the Netherlands. Meanwhile, the Dutch Carnival has started in most southern parts of the Netherlands, with large crowds of people coming together to celebrate. |
| 27-2-2020 | NL | Statistics | A first case of the new coronavirus has been confirmed by the National Institute for Public Health and Environment (RIVM). The patient is a 56 year old man living in Brabant, a southern province of the Netherlands. He most likely contracted the virus on a trip to the Lombardy region in Italy. |
| 28-2-2020 | NL | Statistics | A second case of the coronavirus has been confirmed by the RIVM. The patient is a woman living in Amsterdam, who returned from a trip to the Lombardy region in Italy. |
| 4-3-2020 | NL | Safety measures | The RIVM advises for "only necessary travels" to all of Northern Italy. Many big companies in the Netherlands start to take measures against the virus, even though this is not yet required by authorities |
| 4-3-2020 | International | Safety measures | Italy announces it is closing down schools and universities nationwide until mid-march. |
| 5-3-2020 | NL | Statistics | The amount of positive tested patients in the Netherlands reportedly grew with 41, more than doubling the total amount to 82 patients. According to Bruno Bruins, minister of medical care, this number is due to a catch-up in conducted tests. |
| 6-3-2020 | NL | Statistics | A first patient with COVID-19 dies in the Netherlands. The patient was an 86 year old man who lived in Hoeksche Waard, Zuid-Holland. Total cases grows to: 128. |
| 6-3-2020 | NL | Safety measures | The RIVM advises people in Noord-Brabant to limit their social contacts in case of colds, coughs and fevers. |
| 9-3-2020 | International | Safety measures | Italy's prime minister Guiseppe Conte announces a nationwide forced quarantine on national television. Only people with a valid work or family reason are allowed to travel. All social events are cancelled. |
| 9-3-2020 | NL | Safety measures | Prime minister Mark Rutte holds a nationally televised press conference with Jaap van Dissel, RIVM director of disease control, to inform people on the coronavirus. In this press conference, he advises people to stop shaking hands and to work from home if you live in Noord-Brabant. Earlier that day, the government also advised to regularly wash your hands, cough or sneeze in your elbow and to only make use of paper towels. After the press conference ended, the PM mistakenly shook hands with Jaap van Dissel. |
| 11-3-2020 | International | News | The WHO declares that COVID-19 can be characterized as a pandemic. This does not change the WHO's assessment of the threat posed by the virus, neither does it change what the WHO's actions are towards solving the situation. According to the WHO, the characterization as a pandemic serves to warn countries and to call for countries to 'take urgent and aggressive action'. |
| 12-3-2020 | NL | Statistics | Since yesterday, the total amount of patients that have tested positive has grown with 111, bringing the total amount of cases to 614. The total amount of patients that have died is 5. |
| 12-3-2020 | NL | Safety measures | The RIVM announces that the safety measures implemented in the province of Noord-Brabant now apply to the whole of the Netherlands. Events with over 100 people are banned at least until 31st of March, schools remain open. |
| 14-3-2020 | International | Safety measures | The Spanish government has formally declared a state of emergency over the coronavirus, placing the country in lockdown for the next weeks. |
| 15-3-2020 | NL | Safety measures | In a press conference, minister Bruins and minister of education Arie Slob announces that schools are closed and teachers have to prepare remote teaching for the children at home. Restaurants, bars, sports clubs and more establishments have to close until April 8. People are asked to keep a distance of 1,5 meters to each other where possible. |
| 16-3-2020 | NL | Statistics | Since yesterday, the total amount of patients that have tested positive has grown with 278 and 4 patients have died. This brings the total number of cases to 1.413 and the amount of patients that died to 24. |
| 16-3-2020 | NL | Safety measures | PM Rutte addresses the nation about the coronavirus. In his speech, he announced that the government will not resort to a full lockdown, but instead to opt for a method of controlled spread of the virus. This method aims to build group immunity, but Rutte also stated that a large proportion of the Dutch population will be infected because of this. |
| 16-3-2020 | NL | News | The press conference of PM Rutte has elicited negative reactions among Dutch people, because . There were also positive reactions to the sign language interpreter, because of the gestures she made for the word 'hamsteren' (hoarding). |
| 17-3-2020 | NL | News | In the evening of the 17th, many Dutch people applauded the people with necessary jobs, like care workers, garbage collectors and others. |
| 17-3-2020 | NL | Economy | People have started hoarding certain products, like toilet paper and pasta, from supermarkets around the country. |
| 17-3-2020 | NL | Economy | In a newsletter to the public, the cabinet announces that they will take exceptional economic measures to accommodate the jobs and incomes of self-employed workers, entrepreneurs and large companies. |
| 19-3-2020 | NL | Statistics | Since yesterday, the total amount of patients that have tested positive has grown with 409 and the deaths have grown with 18. This brings the total number of cases to 2.460 and the amount of people that died to 76. |
| 19-3-2020 | NL | News | Bruno Bruins resigns as minister of medical care due to the workload being too taxing. The minister collapsed the previous night during a debate on the coronavirus. Minister Hugo de Jonge takes over his coronavirus-related tasks. |
| 20-3-2020 | NL | News | King of the Netherlands Willem Alexander addressed the nation on national television, telling people that they cannot stop the coronavirus, but they can stop the 'loneliness-virus'. |
| 22-3-2020 | NL | Safety measures | The Dutch government has sent an 'NL-alert' text message to a large proportion of Dutch citizens, telling them to keep 1.5m distance and remain at home if you are sick. |
| 23-3-2020 | NL | Statistics | Since yesterday, the total amount of patients that have tested positive has grown with 573 and the deaths have grown with 43. This brings the total number of cases to 4.204 and the amount of people that died to 179. |
| 23-3-2020 | International | Safety measures | UK Prime Minister Boris Johnson announces that the UK will go into a total lockdown. |
| 23-3-2020 | NL | Safety measures | In a nationally televised press conference, PM Rutte introduces new safety measures, calling it an 'intelligent lockdown'. This means that people are still allowed to go outside as long as they keep 1,5 meters distance. Other new safety measures include that all events are cancelled until June 1st and people are not allowed to meet more than 3 other people outside or in their homes. The government also gave more power to local authorities to hand out fines to people that do not follow the instructions (up to €390 for individuals). |
| 24-3-2020 | NL | Safety measures | In a letter to the house of representatives, the minister of education announces all central final exams in May are cancelled. This is the first time since 1945 that this has happened in the Netherlands. |
| 25-3-2020 | NL | Safety measures | The government made guidelines for responsible shopping to instruct stores on how to keep their businesses running in a way that is safe for both employees and customers. |
| 26-3-2020 | NL | Statistics | Since yesterday, the total amount of patients that have tested positive has grown with 852 and the deaths have grown with 80. This brings the total number of cases to 6.412 and the amount of people that died to 365. |
| 26-3-2020 | NL | News | The Government starts a new campaign that aims to offer people a clear and calming message. The campaign is called 'Alleen samen' (Only together) and is displayed on various media platforms like television, social media, websites. |
| 26-3-2020 | NL | Economy | The government's central planning office (CPB) made four scenarios for the economic impact of the coronavirus pandemic and states that a recession is inevitable. |
| 27-1-1900 | International | News | UK Prime Minister Boris Johnson and Health Secretary Matt Hancock test positive for COVID-19 and stated that they have started to self-isolate. |
| 30-3-2020 | International | Research | Start of data collection of the UK study. |
| 31-3-2020 | NL | Statistics | Since yesterday, the total amount of patients that have tested positive has grown with 884 and the deaths have grown with 93. This brings the total number of cases to 11.750 and the amount of people that died to 864. The RIVM reported that the amount of reported patients is still growing, but growing less hard. |
| 31-3-2020 | NL | Safety measures | In a press conference with Hugo de Jonge, minister of health, Mark Rutte announces that all safety measures initiated on 15 March will be extended until at least 28 April. |
| 1-4-2020 | International | Research | End of data collection of the UK study. |
| 2-4-2020 | NL | Statistics | Since yesterday, the total amount of patients that have tested positive has grown with 1.019 and the deaths have grown with 134. This brings the total number of cases to 13.614 and the amount of people that died to 1.173. |
| 7-4-2020 | NL | Safety measures | In a nationally televised press conference, PM Rutte says that the measures could possibly be loosened a bit after 28 April, however getting back to 'normal' will take considerable time. |
| 9-4-2020 | NL | Statistics | Since yesterday, the total amount of patients that have tested positive has grown with 969 and the deaths have grown with 147. This brings the total number of cases to 20.549 and the amount of people that died to 2.248. |
| 11-4-2020 | NL | News | The Ministry of Health, Welfare and Sport invites companies and experts to contribute ideas on the development and deployment of apps in the fight against the coronavirus. The apps can be used for tracing infections and reporting health problems. |
| 15-4-2020 | NL | News | PM Rutte said in a press conference that the cabinet is considering to loosen safety measures in the near future. This can only happen if the following conditions are met: the pressure on healthcare must be reduced, the elderly must be adequately protected and there must be 'insight into the spread of the virus'. |
| 16-4-2020 | NL | Statistics | Since yesterday, the total amount of patients that have tested positive has grown with 734 and the deaths have grown with 189. This brings the total number of cases to 28.153 and the amount of people that died to 3.134. |
| 20-4-2020 | NL | Research | Start of data collection of the NL study. |
| 21-4-2020 | NL | Statistics | Since yesterday, the total amount of patients that have tested positive has grown with 750 and the deaths have grown with 67. This brings the total number of cases to 33.405 and the amount of people that died to 3.751. |
| 21-4-2020 | NL | Safety measures | In a press conference, PM Rutte announces that schools for children up to 12 can be partially reopened. Children up to 12 are allowed to play organized sports under supervision and children up to 18 can play team sports when keeping a distance. The other initial measures remain until 20 May. All events are cancelled until 1st of September. |
| 21-4-2020 | NL | Research | End of data collection of the NL study. |
| 24-4-2020 | NL | Economy | The ministry of Finance announces that the national budget deficit will be 92 billion euros or 11,8 percent. This is the highest deficit since the second world war. |

## 3.4 Trends

**The United Kingdom - Data collection between March 31th and April 1st, 2020.**Source: https://covid19.who.int/region/euro/country/gb Retrieved on June 10th, 2020.


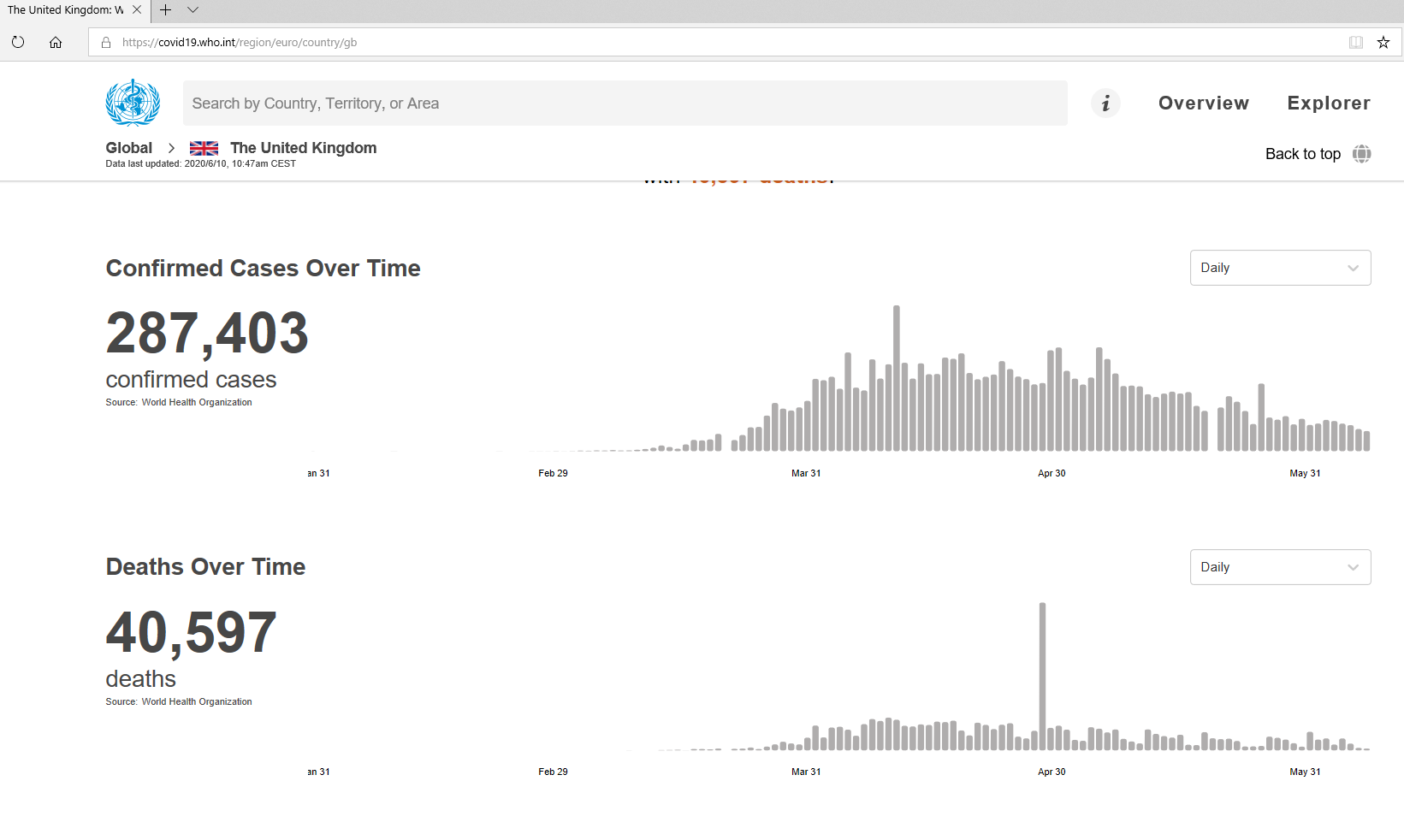


**The Netherlands - Data collection between April 20th and April 21st, 2020.**Source: https://covid19.who.int/region/euro/country/nl Retrieved on June 10th, 2020.


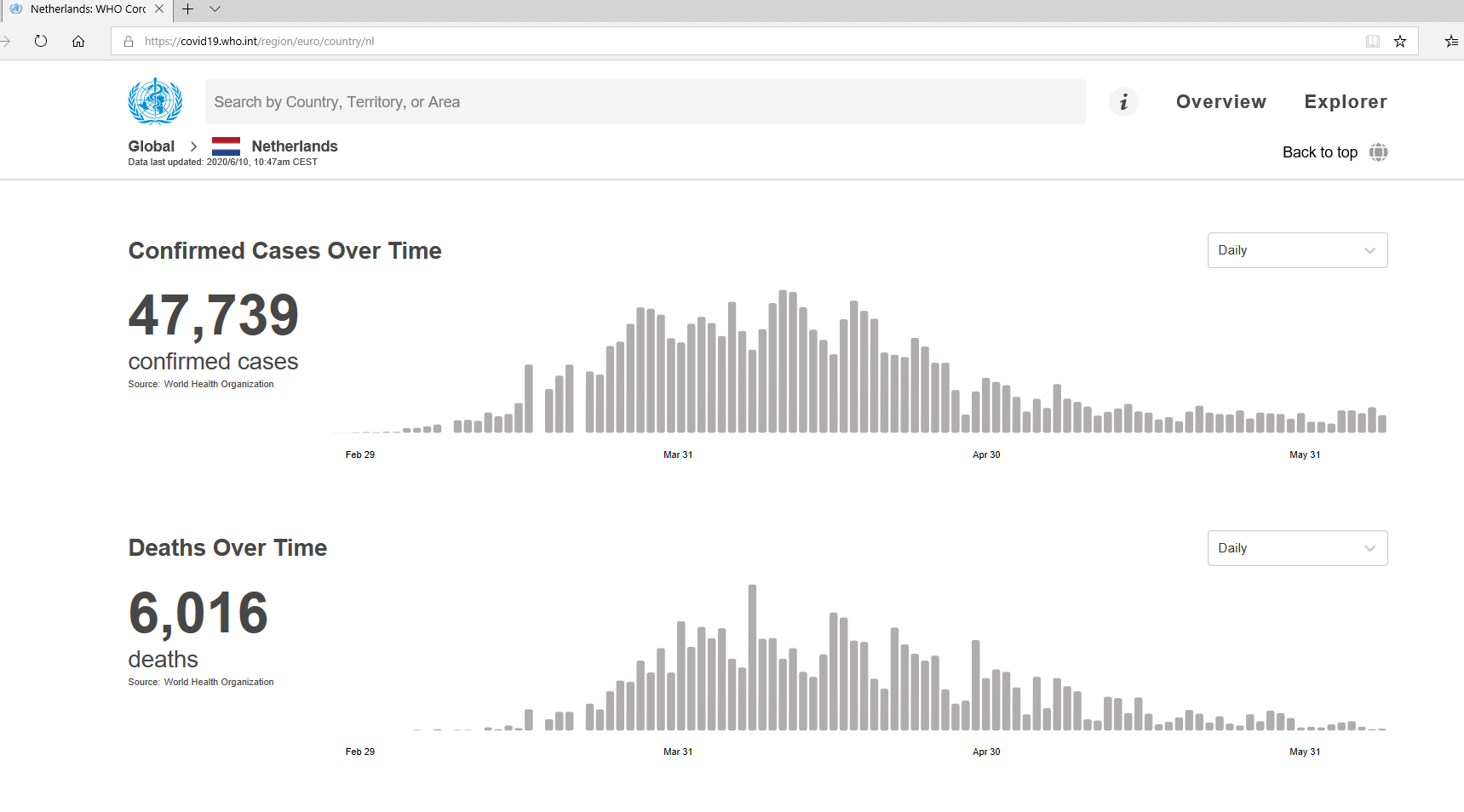


# S4 R script

See ‘S4 Script network analysis COVID-19 comparison study_def’ on OSF: https://osf.io/qc5gb/

# S5 Edge accuracy and edge difference

These PDF files can be found on OSF: https://osf.io/qc5gb/

- Edge accuracy: See ‘S5 Edge accuracy_totalsample’, ‘S5 Edge accuracy_UK’ and ‘S5 Edge accuracy_NL’.
- Edge difference: See ‘S5 Edge difference_totalsample’, ‘S5 Edge difference _UK’ and ‘S5 Edge difference _NL’.
  Interpretation guidance: *a* = .05, black boxes indicate significant differences between edges.

# S6 Centrality stability and centrality difference

These PDF files can be found on OSF: https://osf.io/qc5gb/

- Centrality stability: See ‘S6 Centrality stability_totalsample’, ‘S5 Centrality stability_UK’ and ‘S5 Centrality stability_NL’.
- Centrality difference: See ‘S6 Centrality difference_totalsample’, ‘S5 Centrality difference_UK’ and ‘S5 Centrality difference_NL’.
  Interpretation guidance: *a* = .05, black boxes indicate significant differences between nodes, node strength is presented in the diagonal boxes.

# References

Csardi, G., & Nepusz, T. (2006). The igraph software package for complex network research. *InterJournal*, *Complex Systems*, 1695. http://igraph.org

Dalege, J., Borsboom, D., van Harreveld, F., van den Berg, H., Conner, M., & van der Maas, H. L. J. (2016). Toward a formalized account of attitudes: The Causal Attitude Network (CAN) model. *Psychological Review*, *123*(1), 2–22. https://doi.org/10.1037/a0039802

Dalege, J., Borsboom, D., van Harreveld, F., & van der Maas, H. L. J. (2017). Network Analysis on Attitudes: A Brief Tutorial. *Social Psychological and Personality Science*, *8*(5), 528-537. https://doi.org/10.1177/1948550617709827

Epskamp, S., Borsboom, D., & Fried, E. I. (2018). Estimating psychological networks and their accuracy: A tutorial paper. *Behavior Research Methods*, *50*(1), 195-212. https://doi.org/10.3758/s13428-017-0862-1

Epskamp, S., Cramer, A. O. J., Waldorp, L. J., Schmittmann, V. D., & Borsboom, D. (2012). qgraph: Network Visualizations of Relationships in Psychometric Data. *Journal of Statistical Software*, *48*(4), 1-18. http://www.jstatsoft.org/v48/i04/

Haslbeck, J. M. B., & Waldorp, L. J. (2020). mgm: Estimating Time-Varying Mixed Graphical Models in High-Dimensional Data. *Journal of Statistical Software*, *93*(8), 1-46. https://doi.org/10.18637/jss.v093.i08

Leary, M. R. (1983). A Brief Version of the Fear of Negative Evaluation Scale. *Personality and Social Psychology Bulletin*, *9*(3), 371-375. https://doi.org/10.1177/0146167283093007

R Core Team. (2013). *R: A language and environment for statistical computing.* . In R Foundation for Statistical Computing. http://www.R-project.org/

van Borkulo, C. D., Boschloo, L., Kossakowski, J. L., Tio, P., Schoevers, R. A., Borsboom, D., & Waldorp, L. J. (2017). Comparing network structures on three aspects: A permutation test. https://doi.org/10.13140/RG.2.2.29455.38569.

1. Please note that negative edge weights can appear as positive edge weights in the edge accuracy output. This is because the edge weights are based on networks estimated with mgm in which the sign of the edge weights is stored separately, whereas the edge accuracy analysis is conducted with *bootnet* that does not include the sign from mgm. [↑](#footnote-ref-1)
2. The NCT compares networks of either continuous or binary variables, and not from mixed networks. The results of the NCT (i.e. significant differences between edges) are therefore based on the networks estimated for continuous variables (with EBICglasso). The NCT graph displays those edges that differ significantly according to the results of the NCT with the displayed difference in strength based on the edge weight in the mgm network. [↑](#footnote-ref-2)
3. Statistics/safety measures/news/context/research [↑](#footnote-ref-3)
4. Statistics/safety measures/news/context/research [↑](#footnote-ref-4)
